# Proteomic-Based Discovery of Predictive Biomarkers for Drug Therapy Response and Personalized Medicine in Chronic Immune Thrombocytopenia

**DOI:** 10.1155/2023/9573863

**Published:** 2023-10-31

**Authors:** Samira Gilanchi, Mohammad Faranoush, Mahyar Daskareh, Fatemeh Sadat Sadjjadi, Hakimeh Zali, Alireza Ghassempour, Mostafa Rezaei Tavirani

**Affiliations:** ^1^Proteomics Research Center, Shahid Beheshti University of Medical Sciences, Tehran, Iran; ^2^Pediatric Growth and Development Research Center, Institute of Endocrinology, Iran University of Medical Sciences, Tehran, Iran; ^3^Department of Radiology, Ziaeian Hospital, Tehran University of Medical Sciences, Tehran, Iran; ^4^Department of Tissue Engineering and Applied Cell Sciences, School of Advanced Technologies in Medicine, Shahid Beheshti University of Medical Sciences, Tehran, Iran; ^5^Medicinal Plants and Drugs Research Institute, Shahid Beheshti University, G.C., Evin, Tehran, Iran

## Abstract

**Purpose:**

ITP is the most prevalent autoimmune blood disorder. The lack of predictive biomarkers for therapeutic response is a major challenge for physicians caring of chronic ITP patients. This study is aimed at identifying predictive biomarkers for drug therapy responses.

**Methods:**

2D gel electrophoresis (2-DE) was performed to find differentially expressed proteins. Matrix-assisted laser desorption/ionization time-of-flight mass spectrometer (MALDI-TOF MS) analysis was performed to identify protein spots. The Cytoscape software was employed to visualize and analyze the protein-protein interaction (PPI) network. Then, enzyme-linked immunosorbent assays (ELISA) were used to confirm the results of the proteins detected in the blood. The DAVID online software was used to explore the Gene Ontology and pathways involved in the disease.

**Results:**

Three proteins, including APOA1, GC, and TF, were identified as hub-bottlenecks and confirmed by ELISA. Enrichment analysis results showed the importance of several biological processes and pathway, such as the PPAR signaling pathway, complement and coagulation cascades, platelet activation, vitamin digestion and absorption, fat digestion and absorption, cell adhesion molecule binding, and receptor binding. *Conclusion and Clinical Relevance*. Our results indicate that plasma proteins (APOA1, GC, and TF) can be suitable biomarkers for the prognosis of the response to drug therapy in ITP patients.

## 1. Introduction

Idiopathic thrombocytopenic purpura (ITP) is known as immune thrombocytopenia, or an autoimmune hematological disorder, which is characterized by a decrease in platelet count in peripheral blood and a variety of bleeding symptoms. In severe circumstances, cerebral bleeding can be fatal [1–3]. It is estimated that the prevalence rate of ITP in communities is 2 to 5 in every 100,000 people [4].

The first-line of therapy for treated adult patients with chronic ITP is pharmacotherapy. Glucocorticoids are recommended primarily for the management of ITP patients. Depending on the circumstances, other medicines have also been prescribed as first-line treatments, including intravenous immunoglobulin (IVIG), anti-D (Rho[D] immune globulin intravenous), and rituximab (a monoclonal antibody against the CD20 antigen). Unsuccessful treatment leads to the second-line treatment, which is splenectomy in adults [4–8]. The timing of the procedure for patient management is dependent on the severity of the disease, the physician's decision, the side effects of prior treatment, the amount of physical activity, and the patient's choice [3, 8].

Treatment of chronic refractory ITP is fraught with problems, including that many patients have fewer symptoms, the response to treatment is unclear, and treatments may have adverse side effects [9]. Many different treatments with repeated reports of success have been declared, but the experience of several physicians shows that the responses to treatments are unusual. Also, it is mentioned that recommended treatments mostly have serious and unacceptable side effects [9, 10]. Only 25% of patients have had long-term responses to first-line drug therapy [11]. Besides, side effects with corticosteroids are very common, so frequent injections are tedious, and debilitating headaches after immunoglobulin injections are uncomfortable, they also have an unreliable factor for predicting response to treatment [11, 12]. With the several side effects of pharmacotherapy, a number of patients finally have to undergo splenectomy. To the best of our knowledge, prediction biomarkers for personalized medicine in ITP have not been completely explored. Despite the possible application of platelet autoantibody serologic results in predicting therapy response that was previously discussed [13, 14], it has recently been demonstrated that anti-GPIb/IX antibodies could not predict a patient's nonresponse to glucocorticoids or IVIG [15]. Based on the results of the studies, the current status of predicting biomarkers for personalized medicine in ITP is inconclusive [16]. Thus, identifying predictive biomarkers can be useful in predicting a patient's response to first-line therapy if they are candidates for splenectomy. So it can be valuable in the strategic choice of surgical intervention. Here, we aim to identify plasma biomarkers with specific properties among the human proteome in ITP by applying a combination of high-throughput approaches and computational methods. Based on a proteomic approach, we are able to identify protein alterations in plasma samples of ITP patients that can finally be divided into two categories: positive and negative responses to first-line treatment.

## 2. Materials and Methods

### 2.1. Study Design

In the present study, we first used two-dimensional gel electrophoresis (2-DE) technology to detect differentially expressed proteins in respondents and nonrespondents. Matrix-assisted laser desorption/ionization time-of-flight mass spectrometer (MALDI-TOF MS) analysis is applied to identify differentially expressed protein spots, which are identified by 2-DE in ITP patients with different responses to pharmacotherapy.

**Scheme 1:**
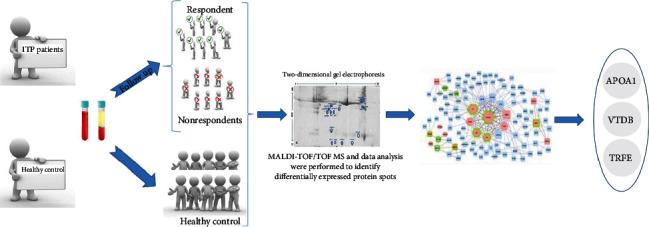
Schematic workflow of the study.

Secondly, we used bioinformatic tools, such as the DAVID Functional Annotation Tool (https://david.ncifcrf.gov/tools.jsp) and Cytoscape (https://cytoscape.org/). DAVID will provide critical information that will help us comprehend the biological themes in our proteomic research. Cytoscape was used to create and visualize PPI networks related to identified proteins to select more important proteins and modules. To confirm the validity of crucial proteins, ELISA (enzyme-linked immunosorbent assay) was performed. ELISA was used to evaluate three identified protein values in plasma samples of three groups (respondents, nonrespondents, and healthy controls).  Scheme 1 represents the workflow of the study.

### 2.2. Patients

Patients were recruited among ITP patients who were referred to the Rasoul Akram Hospital (Tehran, Iran) between January 2018 and February 2019 (Supplementary Table S1). ITP patients were diagnosed using the evidence-based practice guideline for immune thrombocytopenia published by the American Society of Hematology in 2011 [7]. Also, other causes of thrombocytopenia were ruled out. The University Ethics Committee had approved this clinical experiment (Code No. IR.SBMU.RETECH.REC.1399.1234). We collected the plasma samples of ITP patients, including females and males, with an age range of 15-70 years and no previous ITP experience or medication associated with the ongoing disease. Samples were collected before pharmacotherapy for first-line treatment. After three months of medication, patients are divided into two categories: respondents and nonrespondents. Clinical symptoms of the patient, including bleeding, fatigue, and the amount of menstrual bleeding (for females), were the main criteria for classifying respondents and nonrespondents. Platelet count is not the main criteria for classifying respondents and nonrespondents. But for samples with platelet count < 30 × 10^9^/l, they were classified as nonrespondent. Control samples were matched for age and gender.

### 2.3. Plasma Sample Preparation

In order to prepare plasma samples, fresh whole blood samples were collected into EDTA-coated tubes. Samples were transported in dark conditions on ice and centrifuged immediately at 3700 g for 10 min at 4°C two times. The supernatants were separated into 200 *μ*l aliquots and kept at −80°C until subsequent analysis.

We did not use depletion methods to eliminate the most abundant proteins due to concerns that there is not any specific method for depletion [17].

### 2.4. Two-Dimensional Electrophoresis and Image Analysis

Nondepleted plasma samples were analyzed by 2-DE gel electrophoresis. The first dimension was done by using 17 cm IPG isoelectric focusing (IEF) on immobilized pH [3–10] gradient strips. All 2-DE chemicals and Ready-Strip™ IPG strips in this stage were supplied by Sigma Company. Also, Bio-Rad Protean IEF Cell was applied for IEF. The rehydration step was done at 50 V for 12 h with a 500 *μ*g sample of plasma that was premixed with rehydration buffer. Before the second dimension, the equilibration step was done for 30 minutes at room temperature. In the second dimension, protein separation was done based on molecular weight (MW). 12% sodium dodecyl sulfate polyacrylamide gel electrophoresis (SDS-PAGE) gel and buffering systems in the electrophoresis tank (Bio-Rad) were applied for this step. After two-dimensional gel electrophoresis for separating plasma protein, the gels were stained by Coomassie statin (3 L, including 0.3 g Coomassie R250, 18 ml orthophosphoric acid, and 90 ml acetic acid) and then were scanned by a Bio-Rad scanner (GS-800). Then, protein expression changes were analyzed by Progenesis SameSpots software, which compared gels for protein spot detection, evaluating spots' color intensity, spot alignment, and statistical analysis. Fold change (increase or decrease) with 1.5 < value < 1.5 was defined as a cutoff. Also, *p* value < 0.05 was selected as statistically significant differences in spot intensities were identified using one-way ANOVA. Finally, the significant differentially expressed proteins, with increased or decreased expression change from the healthy control gel, were selected to be identified by MALDI-TOF/TOF MS analysis.

### 2.5. In-Gel Digestion and MALDI-TOF MS Analysis

The significantly altered spots in the expression were identified and manually excised from 2-DE gels. In-gel digestion for mass spectrometric characterization was done based on nature protocols [18]. Each sample was treated with Sigma mass grade trypsin that was activated by ammonium bicarbonate overnight at 37°C. Then, 3 *μ*l of each trypsin solution was transferred to new microtubes, and 6 *μ*l of matrix was added (1 : 2 *v*/*v*) in 50% *α*-cyano-4-hydroxycinnamic acid and thoroughly vortexed to generate a homogeneous suspension. A saturated solution of *α*-cyano-4-hydroxycinnamic acid contains 10 ml of matrix in 1 ml of a solution that includes 50% acetone/0.1% and 50% trifluoroacetic acid/0.1% (Agilent Technologies, Palo Alto, USA). Finally, this mixture was used for mass spectrometry analysis. For this aim, 1 *μ*l of this solution was applied directly to a polished steel target plate and allowed to dry in room temperature. Samples were analyzed by an applied Biosystems 4800 MALDI TOF/TOF, and spectra were obtained by an Nd:YAG 200 Hz laser in the reflector positive mode of the mass analyzer. A mass spectrum was recorded after 1200 laser shots. For data interpretation and processing, Explorer Software version 4.0 (Applied Biosystems) was applied.

Peptide mass fingerprints (PMFs) and MS/MS analysis were searched by the Mascot search engine (http://www.matrixscience.com/search_form_select.html) against the SwissProt protein database. The following parameters were utilized in the Mascot search engine: trypsin digestion: maximum one missed cleavage; fixed modification: carbamidomethylation (C); peptide mass tolerance: 1.2 Da; and mass value: [M+H]^+^ and monoisotopic. When the peptide score was above the cutoff value, protein identification was accepted (*p* < 0.05).

### 2.6. Network Construction and Module Selection

Cytoscape version 3.7.2 was applied for interaction analysis. For PPI analysis by Cytoscape, the Human Protein Reference Database (HPRD) and Search Tool for the Retrieval of Interacting Genes (STRING) were selected as interaction sources. HPRD was used as a rich resource of human proteins, which were confirmed experimentally. The interaction of protein-proteins from the HPRD has been widely used by the scientific community [19]. STRING is a metaresource that compiles the majority of the data on protein-protein interactions, scores, and weights [20]. Differentially expressed proteins identified by 2-DE gel were identified by MALDI-TOF MS and searched by HPRD through the BisoGenet app (version 3.0.0). The BisoGenet plugin in Cytoscape software was used to create a PPI network related to altered proteins. So, we selected the HPRD in this plugin for protein-protein interaction. The protein names (UniProt ID) were the input identifiers, and the selected species for these identifiers was Homo sapiens (human) as available in the organism box. In addition, we used the STRING database to visualize the PPI network related to altered proteins. Then, we merged these two networks and analyzed this new network to select a more important proteins and modules. One of the assessed network criteria was the type of network, indicating whether it belongs to a random or scale-free network. Biological networks are scale-free. Scale-free means that few nodes have a high degree (“balls”), and most nodes have a low degree and are only related to one or a few neighbors [21]. In network analysis, based on two cutoff criteria, the topologically important proteins were selected. The first criterion to select central protein was the hub. The selection of hub threshold is calculated based on the following formula: the mean + 2 × standard deviation (mean + 2 × SD) of the degree. Next, nodes with a degree ≥ threshold were selected as hubs. In the second step, the bottlenecks were chosen from the top 10% of nodes with the highest betweenness centrality [22]. Bottlenecks, as they facilitate the flow of information between densely connected subnetworks (modules), play a key role in mediating linkage inward a certain network [23]. The integration of these two parameters was called “hub-bottleneck” which are critical nodes for network integrity [24]. Then, Molecular Complex Detection (MCODE) was used to screen modules. In a PPI network, subgraphs of highly interconnected proteins can be considered as protein complexes or functional modules. The MCODE plugin is used to determine functional modules in a network that finds highly connected regions in PPI networks that may display molecular complexes [25, 26].

### 2.7. Functional and Pathway Enrichment Analyses

Proteomic investigations generate a lot of data. Also, simply identifying and quantifying proteins from a cell's proteome or subproteome is insufficient to fully comprehend complicated biological mechanisms. In order to comprehend the results of high-throughput proteomics, functional annotation analysis of protein datasets using bioinformatic techniques is required [27].

The DAVID (Database for Annotation, Visualization and Integrated Discovery) [28] was employed to enrich the proteomic and PPI network data. Enrichment analysis was conducted to clarify the Kyoto Encyclopedia of Genes and Genomes (KEGG) pathway [29] and Gene Ontology that affect the type of response to drug therapy in patients with chronic ITP. Gene Ontology (GO) analysis includes cellular components, molecular functions, and biological processes [30]. Statistical significance was defined as *p* < 0.05 for both the GO and KEGG enrichment analyses.

### 2.8. Enzyme-Linked Immunosorbent Assay (ELISA)

The sandwich ELISA was used to validate key proteins that were selected by network analysis. For this aim, plasma samples from three groups (respondent, nonrespondent, and healthy controls) were analyzed. ELISA kits were purchased from Zell Bio Co. (Veltlinerweg 29, 89075 Ulm, Germany). ELISA kits were used to determine the concentrations of identified proteins in plasma, as indicated by the manufacturer.

### 2.9. Statistical Analysis

Statistical analysis of 2-DE gels was done by the Progenesis SameSpots software (Nonlinear Dynamics, USA). To determine the statistically significant changes, ANOVA and fold changes were used. Protein spots with a *p* value < 0.05 and a fold change ≥ 1.5 were regarded as having changed in protein expression significantly. The ELISA data were analyzed using the GraphPad Prism software application, version 6 (GraphPad Software, Inc., La Jolla, CA, USA). One-way ANOVA and Tukey's multiple comparison test were used to analyze the data. A statistically significant difference was defined as a *p* value < 0.05.

## 3. Results

The aim of this study is to find molecular signatures that help us discriminate between drug respondent and nonrespondent patients with chronic type ITP. After following the patients, we found that twenty patients were diagnosed with newly diagnosed ITP, and sixteen patients suffered from chronic ITP (lasting for more than 12 months). All sixteen chronic ITP patients were followed up for drug responses. Samples were divided into two groups: respondents and nonrespondents. In each category, there were eight patients. Four patients were excluded. Ten normal people, five males and five females, were included in the control groups.

### 3.1. Sixteen Proteins Were Identified 2D-Electrophoresis and MALDI-TOF/TOF-MS Analysis

**Figure 1:**
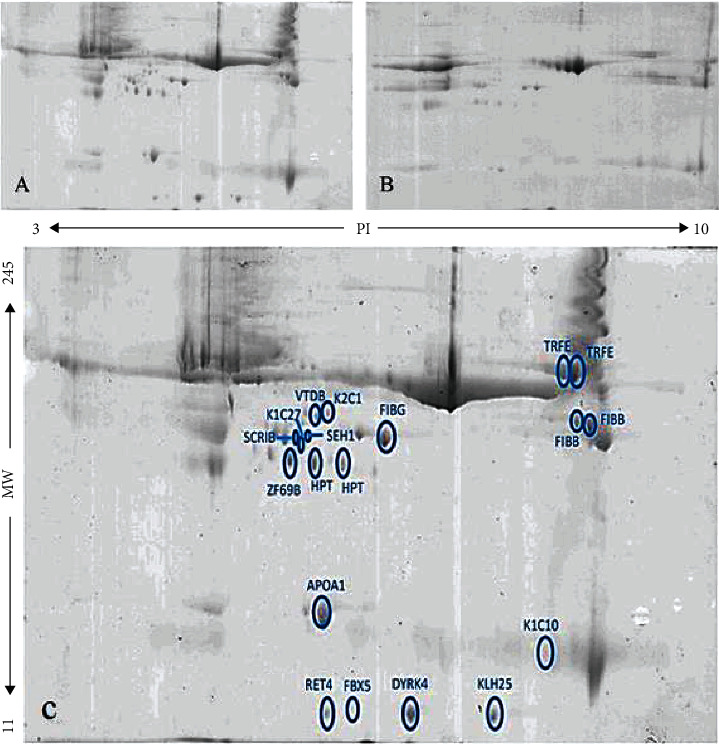
The protein profile of chronic ITP samples separated by two-dimensional gel electrophoresis (2-DE): (A) respondents; (B) nonrespondents. (C) Nineteen differentially expressed protein spots identified by Progenesis SameSpots software (2-DE image analysis).

The plasma of each group of samples (respondents and non-respondents) was pooled, and their proteins were separated by 2-DE. All 2-DE gel experiments were repeated three times to minimize experimental variations. Figures 1(a) and 1(b) represent Coomassie Blue-stained 2-DE gel images for each group of samples.

Progenesis SameSpots analysis that showed nineteen differentially expressed protein spots provides a comprehensive insight into response to first-line treatment (pharmacotherapy) of ITP patients (Supplementary Figure S1 and Supplementary Table S2). The 2-DE gel of the healthy group was used to understand the upregulation or downregulation that was occurred in the differentially expressed spots between respondents and nonrespondents (Supplementary Figure S2). The comparison of the mass of these differentially expressed spots with the control group showed that eighteen protein spots were found to be downregulated in nonrespondents while one protein spot was upregulated. These protein spots were extracted from the gels and prepared for analysis by MALDI-TOF MS/MS. Then, SwissProt database by using the Mascot search engine program was used to identify proteins. Sixteen proteins were identified, including K2C1, VTDB, FIBB (two spots known as FIBB), FIBG, ASCL1, HPT (two spots known as HPT), APOA1, K1C10, FBX5, RET4, IGDC4, KLH25, RL31, SCRIB, SEH1, and TRFE (two spots known as TRFE) (Figure 1(c) and Table 1). The spectra and sequences of nineteen proteins which are identified by MALDI-TOF MS/MS are shown in supplementary Figure S3 and S4.

### 3.2. TRFE, APOA1, and VTDB Are Hub-Bottlenecks in Protein-Protein Interaction Network

**Figure 2:**
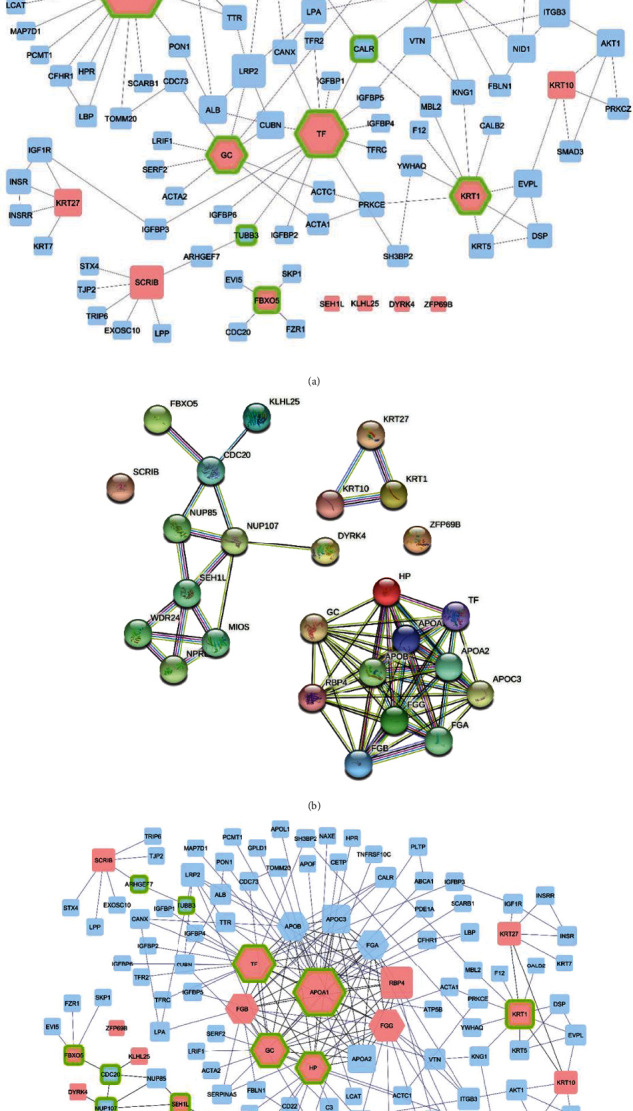
(a) Protein-protein interaction network of 16 differentially expressed proteins in respondents and nonrespondents from HPRD source. The PPI network consists of 103 nodes and 137 edges. Pink nodes: 16 differentially expressed proteins identified by proteomic approaches. Blue nodes: nodes added by the HPRD in Cytoscape software. Hexagon nodes: hub proteins with the highest degree. Green border nodes: bottleneck nodes with the highest betweenness centrality. (b) Protein-protein interaction network of 16 query proteins which are related to drug treatment response of chronic ITP patient from STRING database source. The PPI network consists of 26 nodes and 73 edges. (c) Merged PPI network from HPRD and STRING database source. The PPI network consists of 110 nodes and 137 edges. The size of the nodes is adjusted based on the degree. Pink nodes: 16 differentially expressed proteins identified by proteomic approaches. Blue nodes: nodes added by the HPRD in Cytoscape software. Hexagon nodes: hub proteins. Green border nodes: betweenness centrality.

A PPI network of differentially expressed proteins was established based on the HPRD integrated into Cytoscape. The network analysis displayed 103 nodes and 137 edges. The network was analyzed by network analysis, and the correlation rate of network nodes was calculated to be 0.971 (Figure 2(a)). Sixteen differentially expressed proteins were mapped to the STRING database to acquire protein-protein interaction (PPI) networks (Supplementary Figure S5). By using the more option twice to have more protein connections from this database, the number of nodes reached 26 (Figure 2(b)). Then, the PPI network obtained from the STRING database was integrated with the HPRD network using Cytoscape software, which merged networks consisting of 110 nodes and 206 edges. The correlation rate of the new network nodes was improved and reached 0.985 (Figure 2(c)), and the network is scale free. To select a hub protein, nodes were graded based on degree scores and the nodes with the highest connections were selected as hubs. In this study, for merged network, cutoff 14 was selected as a hub. Also, nodes with the highest betweenness centrality were defined as bottlenecks. Accordingly, four differentially expressed proteins identified from proteomic analysis were selected as hub-bottlenecks for the integrated network (TRFE, APOA1, VTDB, and HP) (Table 2). Also, four differentially expressed proteins identified from proteomic analysis were selected as hub-bottlenecks for the network which was established by HPRD (TRFE, APOA1, VTDB, and K2C1) (Supplementary Table S3). Therefore, three common proteins in both networks were confirmed as hub-bottlenecks, including TRFE, APOA1, and VTDB.

**Figure 3:**
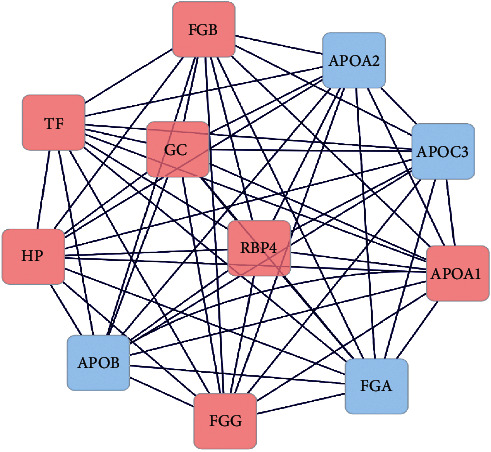
The most important modules involved in the PPI network. The pink nodes represent the input genes to the network, and the blue nodes represent the genes added by databases. The edges indicate the connection between two genes.

Then, the MCODE plugin is used to determine functional modules in the network. The cutoff threshold was chosen to be >4. Based on the cutoff threshold, one module was identified by MCODE for the merged network, including 11 nodes and 56 edges that consist of GC, APOB, RBP4, APOA1, TF, APOA2, FGG, APOC3, FGA, FGB, and HP (Figure 3). This important module includes all network hubs. Also, seven protein spots that were selected by the 2-DE gel as differentially expressed proteins exist in this module (Table 3).

### 3.3. Functional and Pathway Enrichment Changes in Respondent/Nonrespondent

DAVID was used to undertake functional and pathway enrichment analyses to learn more about the function of identified altered proteins discovered by 2-DE gel. The results of the GO analysis are presented from three aspects, including biological processes (BP), molecular functions (MF), and cellular components (CC) (Supplementary Table S4). They were involved in seventeen significant biological processes. The significant biological processes were associated with platelet degranulation, blood coagulation, fibrin clot formation, vitamin transport, blood coagulation, fibrin clot formation, and positive regulation of substrate adhesion-dependent cell spreading. The significant molecular functions involved in two categories include structural molecule activity and protein binding. The significant cellular components related to altered proteins are contained in twelve categories. The most significant cellular components is involved in the blood microparticle, extracellular space, and fibrinogen complex. The five most significant GO terms are shown in Table 4. No KEGG pathway was significant for proteomic data.

Then, we used GO enrichment and KEGG pathway analyses to extract biological information from the merged PPI network nodes. GO enrichment analysis data are represented in Supplementary Tables S5-7. The functional enrichment analysis revealed that these genes are engaged in fourteen significant pathways, according to *p* values < 0.05, such as complement and coagulation cascades, vitamin digestion and absorption, PPAR signaling pathway, platelet activation, Rap1 signaling pathway, fat digestion and absorption, hematopoietic cell lineage, and cell cycle (Table 5).

Also, GO and pathway enrichment analyses of modules were performed. We identified the pathways of the module, which includes all network hubs, and showed the importance of five pathways. As shown in Table 6, this module is enriched in PPAR signaling pathways, complement and coagulation cascades, platelet activation, vitamin digestion and absorption, and fat digestion and absorption. These enrichment data showed the most important pathways for responding to drug treatment in ITP patients. Moreover, Gene Ontology terms related to MF and BP were overrepresented in module proteins with significant *p* values, primarily involved in functions associated with cholesterol, lipoprotein and phospholipids, cell adhesion molecule binding and receptor binding, platelet degranulation aggregation and activation, vitamin transport, and apoptotic process (Supplementary Table S8). The enrichment results of this important module confirmed the enrichment results of the differentially expressed proteins. These results indicate the importance of these biological functions in the type of response of ITP patients to drug therapy.

### 3.4. Validation of Downregulation of APOA1, VTDB, and TRFE by ELISA in Plasma of Nonrespondent

**Figure 4:**
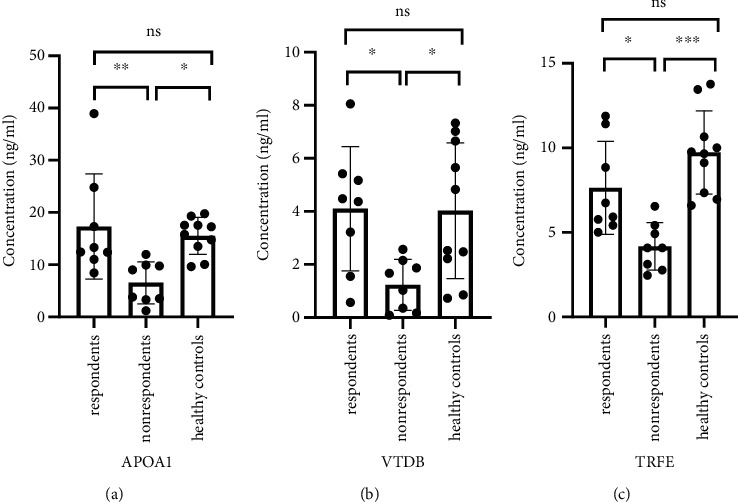
The plasma level of three hub proteins was validated by ELISA. One-way ANOVA analysis and Tukey's multiple comparison test showed significant different expression levels of three hub proteins among the three groups (respondents (*n* = 8), nonrespondents (*n* = 8), and healthy controls (*n* = 10)). The plasma levels of each three proteins included (a) APOA1, (b) VTDB, and (c) TRFE in nonrespondents which were significantly lower than that of respondents and healthy controls. ^∗^*p* < 0.05, ^∗∗^*p* < 0.01, and ^∗∗∗^*p* < 0.001; ns: nonsignificant.

In two networks constructed by the HPRD and a merged network (the HPRD and STRING databases), four proteins were selected as hub-bottleneck proteins. Three common proteins in both methods were selected as hub-bottleneck proteins. To validate the results of the proteomic and network analysis data, we further determined the expression levels of these three common proteins (APOA1, VTDB, and TRFE) in plasma samples by the ELISA technique. All three proteins were separately analyzed by ELISA in plasma samples from 8 respondents, 8 nonrespondents, and 10 controls. Statistical analysis (one-way ANOVA and Tukey's multiple comparison test) was done by applying GraphPad Prism software v.5.04 (GraphPad Software, San Diego, CA). Importantly, analysis of ELISA results indicates that the plasma concentrations of these three proteins significantly decreased in nonrespondents compared to the respondents and control groups. However, there is no significant difference between the respondent and control groups (*p* > 0.05) (Figure 4).

The plasma concentration levels of APOA1 in respondents, nonrespondents, and healthy controls were 18.03571 ± 10.6335 ng/ml, 6.302083 ± 3.672867 ng/ml, and 16.18519 ± 3.047306 ng/ml, respectively. The results of Tukey's multiple comparison test indicated that the plasma levels of APOA1 in the nonrespondents were significantly lower than those in the respondents (*p* < 0.001) and healthy controls (*p* < 0.01). Plasma levels of VTDB in responsive patients and healthy controls did not differ significantly (*p* > 0.05). The plasma levels of APOA1 in respondent versus nonrespondent, nonrespondent versus healthy controls, and respondent versus healthy controls were 95% CI 3.444 to 20.02 ng/ml, -17.67 to -2.100 ng/ml, and -6.222 to 9.923 ng/ml, respectively.

VTDB protein was quantitated by ELISA in respondent, nonrespondent, and healthy control samples. The concentration levels of VTDB in plasma samples of respondents, nonrespondents, and healthy controls were 4.064286 ± 2.529134 ng/ml, 1.235417 ± 0.962695 ng/ml, and 4.383333 ± 2.440059 ng/ml, respectively.

The plasma protein level of VTDB was examined by Tukey's multiple comparison test. The results demonstrated that VTDB levels in the nonrespondents were significantly lower than those in the respondents and healthy controls (*p* < 0.01). No significant difference was observed between plasma levels of VTDB in respondents and healthy controls (*p* > 0.05). The plasma levels of VTDB in respondent versus nonrespondent, nonrespondent versus healthy controls, and respondent versus healthy controls were 0.08960 to 5.568 ng/ml, -5.720 to -0.5761 ng/ml, and -2.986 to 2.348 ng/ml.

The abundance of TRFE protein in plasma samples was also measured by ELISA. The results of ELISA revealed that the concentrations of TRFE in plasma samples of respondents, nonrespondents, and healthy controls were 7.752747 ± 2.944662 ng/ml, 3.835165 ± 1.10778 ng/ml, and 10.24786 ± 2.665811 ng/ml, respectively.

The plasma levels of TRFE were evaluated by Tukey's multiple comparison test. The results showed that the nonrespondents were significantly lower than those in the respondent (*p* < 0.01) and healthy controls (*p* < 0.0001). Between the respondents and the healthy controls, there was no significant difference in TRFE levels (*p* > 0.05). The plasma levels of VTDB in respondent versus nonrespondent, nonrespondent versus healthy controls, and respondent versus healthy controls were 0.6573 to 7.178 ng/ml, -9.487 to -3.339 ng/ml, and -5.569 to 0.5787 5 ng/ml.

## 4. Discussion

Human plasma is known as the largest and richest species in each proteome sample and is considered to have more attention for disease study. Many scientific evidence indicated that alteration in the abundance or structures of plasma proteins resulted in many human diseases [31, 32].

Among the various diseases, it is clear that clinicians still face a number of challenges in caring for autoimmune patients, including (a) accurate diagnosis as soon as possible, (b) reliable classification of patients at risk by predicting outcomes to prevent disease progression, and (c) monitoring the titer of dosage and therapeutic response during the course of the disease in order to stimulate timely drug intervention [33]. Therefore, given the problems in diagnosing the type of response of patients to drug therapy, comprehensive analysis of plasma to find biomarkers that indicate the status of response to drug therapy in patients is very important.

The present comparative proteomic approach based on 2-DE and MALDI-TOF-MS identified nineteen changed protein spots in two groups of chronic ITP patients with positive and negative responses to drug treatment. Network analysis showed three more important spots. The ELISA results established that the data taken from this study is valuable.

Our proteomic studies and network analysis demonstrated the importance of vitamin D-binding protein (VTDB) for drug response in chronic ITP patients. The ELISA also confirmed the decrease in plasma expression of this protein in nonrespondents.

In 2014, a study by van Bladel et al. indicated that platelet function was directly related to the bleeding phenotype in patients with ITP. The results of their studies showed a decrease in platelet degranulation as an important factor in the severity of the disease [34]. The results of the current study also showed a decrease in the expression of FIBG, TRFE, and APOA1 proteins and the elimination of FIBB in patients who showed a negative response to drug therapy. The analysis of biological processes showed that these proteins are important in platelet degranulation. Since APOA1 and TRFE were identified as hub-bottlenecks in the network, ELISA results also confirmed the decrease in the expression of these two proteins in the plasma of patients who showed a negative response to drug therapy. These findings do not support the findings of Illes et al., who stated that platelet degranulation is not significant in ITP [35]. Based on a study (Bastos-Amador P et al., 2012), which reported a detailed proteomic study on plasma microvesicles (MVs), there is considerable variation in MV protein composition in blood plasma, which may have important therapeutic consequences. Proteomic analysis of circulating MVs from healthy donors indicated the importance of APOA1 as a carrier and solute transporter in healthy blood plasma. The results of our study also showed that this protein is downregulated in nonrespondent patients [36].

Čulić et al. (2016) discovered the role of vitamin D in chronic ITP in a study on serum vitamin D levels in pediatrics. The results of their studies revealed that hypovitaminosis D may have a role in the development of chronic types of ITP by causing immunological abnormalities, and VD supplementation by modulating the immune system may reduce the risk of chronic disease [37]. In 2018, other evidence on the significance of vitamin D in ITP and several other autoimmune diseases by Čulić pointed to the high importance of vitamin D in these diseases and wrote, “Unfortunately, the drug has many problems related to immune system diseases.” It is hoped that future research will show how important it is to compensate for VD deficiency to prevent, reactivate, and degrade immune diseases [38]. The current results indicate the critical role of VTDB in the drug treatment of adults with the chronic form of ITP, which is involved in transporting and storing vitamin D.

Mouabbi et al. revealed that ITP patients with iron deficiency did not respond to steroids, which are an effective first-line therapy for ITP. They treated patients with a single infusion of iron dextran to replenish their iron stores [39]. Also, our finding confirmed the importance of iron in response to drug therapy. Our study revealed that TRFE was downregulated in nonrespondent patients, and this protein is a transferrin. Transferrins are iron-binding transport proteins. It is responsible for the transport of iron from sites of absorption and heme degradation to those of storage and utilization.

The results of Zhang et al.'s study showed the importance of the PPAR pathway in ITP disease. Their findings examining self-healing patients and the chronic type of the disease relied on the role of oxidative stress in the pathogenesis of chronic ITP and suggested that factors that improve oxidative stress (especially in the early stages of the disease) or modulate the PPAR pathway should be noticed as novel therapeutic options [40]. Among the significant pathways, we observed that the PPAR pathway plays a crucial role, and it was also demonstrated as the most valid pathway for the significant network module.

The results of our proteomic analysis showed a decrease in plasma haptoglobin protein expression in patients who responded negatively to drug therapy. The results of the network analysis also introduced this protein as a hub-bottleneck protein. Data obtained from Zheng et al.'s study showed the importance of this protein in response to splenectomy treatment. Their findings imply that serum Hp levels may be a good predictor of long-term splenic retinopathy response in ITP and may help researchers better understand the pathophysiological distinctions between respondents and nonrespondents. The results of their 2D gel electrophoresis analysis showed a decrease in the expression of this protein in patients who did not show a long-term response to treatment [41]. Also, data obtained from the samples of patients in the Salama et al. study indicated that the haptoglobin level plays an important role in the patients' condition and bleeding rate [42]. This data is consistent with our observations.

Our bioinformatic analysis of identified proteins upholds the importance of the complement pathway in ITP, in agreement with the results of Kurata et al.'s study. They reported that in most chronic ITP patients, *in vivo* activation of complement (C) occurs by binding C3 and C9 to the platelet surface. This type of C activation may lead to more effective phagocytosis (C3b) and presumably platelet lysis (C5-9) in some patients with ITP [43]. Also, according to the study of Martin and Chan [44], the efficacy of IVIG in a small number of autoimmune disorders, such as ITP and MG, implies that the contributions of antibodies, complement mechanisms, and FcRs may play a role in B cell-mediated autoimmune diseases. Our study's findings might support the Martin and Chan study, which revealed the role of complement pathways in the effectiveness of IVIG in ITP patients. Positive responses to IVIG treatment in the respondent group might be associated with activation of the complement pathway because their related proteins were downregulated in nonrespondent patients.

The results of our studies, as well as metabolic studies and network analysis by Li et al., who studied the therapeutic effects of Zi Dian Fang, a multitarget traditional Chinese medicine (TCM), demonstrated the importance of platelet proliferation pathways. They addressed the hematopoietic cell lineage, coagulation cascade and complement, and the JAK-STAT signaling pathway involved in platelet proliferation pathways [45]. The results of enrichment analysis of the respondent and nonrespondent groups to drug therapy also represented three pathways related to platelet proliferation, including hematopoietic cell lineage, coagulation and complement, and platelet activation. Three of the proteins identified by 2D electrophoresis and mass spectrometry, including FIBB, FIBG, and K2C1, are active in this pathway. In addition, the results of biological processes and KEGG pathway analysis showed the critical role of complement in the type of response to treatment. The results of proteomic evidence by Liu et al., on patients' plasma showed the importance of platelet activation. Using bioinformatic studies and KEGG pathway analysis on proteomic data, which is obtained by the LC MS/MS method, they showed that platelet activation plays a crucial role in this disease [46]. Our data also reported the importance of this pathway in ITP by examining the protein-protein network-related pathways of chronic patients who responded positively and negatively to drug therapy. Two-dimensional gel electrophoresis analysis also showed a change in the expression of FIBB and FIBG proteins, which are involved in platelet activation.

Data obtained from bioinformatic analysis by Zuo et al. demonstrated the importance of the Hippo signaling pathway. They examined the plasma miRNA profile of patients with ITP by microarray. Following that, bioinformatic analysis was used to assess potential molecular networks that might be regulated by the discovered miRNAs. Analysis of miRNA-associated pathways by KEGG showed the association of the Hippo signaling pathway with a very valid *p* value [47]. Furthermore, Liu et al.'s proteomic analysis revealed a decrease in the expression of proteins related to the Hippo signaling pathway and its role in this disease. Their evidence addressed the importance of protein 14-3-3 (YWHAH), which is a protein linked to apoptosis. KEGG pathway analysis showed that it is active in the Hippo signaling pathway and cell cycle pathways [46]. Here, to shed light on the mechanisms in drug response of chronic ITP patients, the apoptosis pathway and cell cycle were highlighted. Our study was demonstrated by examining the pathways related to protein-protein network proteins retrieved from Cytoscape. Also, SCRIB, FIBB, and FIBG proteins, which were identified by gel electrophoresis and mass spectrometry, are among the proteins involved in apoptosis, verifying the implication of this biological pathway in ITP and the type of response to drug therapy.

In conclusion, we identified nineteen plasma proteins by 2-DE that were differentially expressed in respondents and nonrespondents to drug therapy. By Cytoscape analysis, a panel of three more essential proteins (APOA1, VTDB, and TRFE) exhibited predictive value in the prognosis of ITP patients' responses to drug treatment. These findings could be crucial in offering new insights into the pathogenic mechanisms of ITP and would be helpful in understanding the pathophysiological variations between respondents and nonrespondents to drug treatment.

While we are concluding the potential biomarkers for predicting drug therapy response in ITP patients, the plasma levels of APOA1, VTDB, and TRFE were shown to be positively correlated with response to the first-line therapy of ITP patients, but further validation and investigation using larger patient cohorts are necessary before clinical relevance. Also, more investigation into the biology of APOA1, VTDB, and TRFE could be helpful in revealing new understandings of the pathophysiological differences between respondents and nonrespondents. Our study had some limitations, including accessibility to precise techniques and an appropriate sample size. Applying bottom-up proteomics, such as the shotgun strategy for discovering new biomarkers, and using a relative quantitation method like isotope-coded affinity tags (ICAT) to accurately quantify proteins to compare protein or peptide abundance between samples and validate them in large-scale population cohorts with targeted proteomics can be helpful.

## Figures and Tables

**Table 1:** The list of identified nineteen protein spots with some data inferred from 2-DE gel image analysis and Mascot based on SwissProt database (*p* < 0.05).

	Mascot results	Gene name	Mascot score	Expression changes on 2-DE gel	Fold changes
	*Downregulated*				
1	K2C1_HUMAN	KRT1	73	Downregulated in nonrespondents	3.7
2	VTDB_HUMAN	GC	70	Downregulated in nonrespondents	1.7
3	FIBB_HUMAN	FGB	115	Downregulated in nonrespondents	2
4	FIBB_HUMAN	FGB	120	Downregulated in nonrespondents	3.7
5	FIBG_HUMAN	FGG	144	Downregulated in nonrespondents	1.7
6	ZF69B_HUMAN	ZFP69B	38	Downregulated in nonrespondents	1.9
7	HPT_HUMAN	HP	127	Downregulated in nonrespondents	1.7
8	HPT_HUMAN	HP	123	Downregulated in nonrespondents	2.6
9	APOA1_HUMAN	APOA1	174	Downregulated in nonrespondents	3.1
10	FBX5_HUMAN	FBXO5	43	Downregulated in nonrespondents	2.1
11	RET4_HUMAN	RBP4	72	Downregulated in nonrespondents	5.4
12	DYRK4_HUMAN	DYRK4	35	Downregulated in nonrespondents	3.8
13	KLH25_HUMAN	KLHL25	42	Downregulated in nonrespondents	4.1
14	SCRIB_HUMAN	SCRIB	48	Downregulated in nonrespondents	2.2
15	K1C27_HUMAN	KRT27	42	Downregulated in nonrespondents	2.4
16	SEH1_HUMAN	SEH1L	34	Downregulated in nonrespondents	2.6
17	TRFE_HUMAN	TF	183	Downregulated in nonrespondents	1.6
18	TRFE_HUMAN	TF	141	Downregulated in nonrespondents	1.7
	*Upregulated*				
19	K1C10_HUMAN	KRT10	118	Upregulated in nonrespondents	2

**Table 2:** The list of hubs and bottlenecks and hub-bottlenecks of merged network.

	Gene name	Degree	Betweenness centrality
*Hubs*			
1	APOA1	31	0.36595308
2	TF	23	0.40234675
3	GC	19	0.16563324
4	HP	17	0.13375361
5	FGG	17	0.11910425
6	FGB	15	0.06095966
7	FGA	14	0.05532558
8	APOB	14	0.02554443
*Bottlenecks*			
1	CDC20	4	0.59090909
2	FBXO5	4	0.45454545
3	NUP107	5	0.43181818
4	TF	23	0.40234675
5	APOA1	31	0.36595308
6	SEH1L	5	0.18939394
7	GC	19	0.16563324
8	TUBB3	2	0.13796193
9	HP	17	0.13375361
10	KRT1	11	0.1318414
11	ARHGEF7	2	0.11959686
*Hub-bottlenecks*			
1	APOA1	31	0.36595308
2	TF	23	0.40234675
3	GC	19	0.16563324
4	HP	17	0.13375361

**Table 3:** Detected modules in the merged network (HPRD and STRING) by MCODE plugin.

Cluster	Topology of module	Number of nodes	Number of edge	Score	Input proteins to PPI networks	Proteins presented in the modules
1	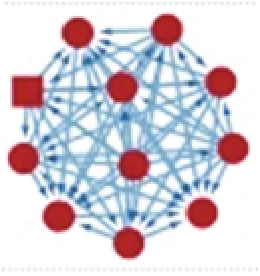	11	56	11	APOA1, TF, GC, FGG, HP, FGB, RBP4	HP, FGB, FGA, APOC3, FGG, APOA2, TF, APOA1, RBP4, APOB
2	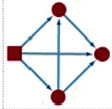	4	6	4	KRT1	KRT5, EVPL, KRT1, DSP
3	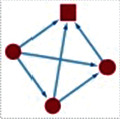	4	6	4	SEH1L	MIOS, NPRL2, SEH1L, WDR24

**Table 4:** The more significant enriched GO analysis of sixteen differentially expressed proteins.

	Category	Term	Count	*p* value	Gene symbol
	*Biological process*				
1	GOTERM_BP_DIRECT	GO:0002576~platelet degranulation	4	9.67*E*-05	FGB, TF, FGG, APOA1
2	GOTERM_BP_DIRECT	GO:0042730~fibrinolysis	3	1.55*E*-04	FGB, KRT1, FGG
3	GOTERM_BP_DIRECT	GO:1900026~positive regulation of substrate adhesion-dependent cell spreading	3	3.64*E*-04	FGB, FGG, APOA1
4	GOTERM_BP_DIRECT	GO:0072378~blood coagulation, fibrin clot formation	2	0.003569	FGB, FGG
5	GOTERM_BP_DIRECT	GO:0051180~vitamin transport	2	0.004459	APOA1, GC
	*Cellular component*				
1	GOTERM_CC_DIRECT	GO:0072562~blood microparticle	7	1.43*E*-09	FGB, TF, KRT1, FGG, HP, APOA1, GC
2	GOTERM_CC_DIRECT	GO:0005615~extracellular space	9	3.51*E*-06	FGB, TF, RBP4, KRT1, FGG, HP, APOA1, KRT10, GC
3	GOTERM_CC_DIRECT	GO:0070062~extracellular exosome	11	1.07*E*-05	FGB, TF, RBP4, KRT27, KRT1, FGG, HP, APOA1, SCRIB, KRT10, GC
4	GOTERM_CC_DIRECT	GO:0005576~extracellular region	7	0.00117	FGB, TF, RBP4, FGG, HP, APOA1, GC
5	GOTERM_CC_DIRECT	GO:0005577~fibrinogen complex	2	0.007385	FGB, FGG
	*Molecular function*				
1	GOTERM_MF_DIRECT	GO:0005198~structural molecule activity	4	1.00*E*-03	FGB, KRT27, KRT1, FGG
2	GOTERM_MF_DIRECT	GO:0005515~protein binding	12	0.0401263	FGB, DYRK4, TF, RBP4, SEH1L, KRT1, FGG, HP, ZFP69B, APOA1, SCRIB, FBXO5

**Table 5:** The significant enriched KEGG pathway analysis for 110 networks nodes.

	Category	Term	Count	*p* value	Genes
1	KEGG_PATHWAY	hsa04610: complement and coagulation cascades	10	1.07*E*-08	FGB, C3, FGA, F12, FGG, F13A1, F13B, KNG1, SERPINA5, MBL2
2	KEGG_PATHWAY	hsa04145: phagosome	10	8.68*E*-06	C3, SCARB1, ITGAM, TFRC, TUBB3, ITGB3, CANX, ITGB2, CALR, MBL2
3	KEGG_PATHWAY	hsa05150: Staphylococcus aureus infection	6	1.32*E*-04	C3, ITGAM, ITGB2, FGG, ICAM1, MBL2
4	KEGG_PATHWAY	hsa04977: vitamin digestion and absorption	4	0.001043	SCARB1, CUBN, APOA1, APOB
5	KEGG_PATHWAY	hsa05143: African trypanosomiasis	4	0.003434	HBB, APOA1, HPR, ICAM1
6	KEGG_PATHWAY	hsa04975: fat digestion and absorption	4	0.005529	ABCA1, SCARB1, APOA1, APOB
7	KEGG_PATHWAY	hsa04611: platelet activation	6	0.00698	FGB, FGA, ITGB3, FGG, AKT1, PRKCZ
8	KEGG_PATHWAY	hsa04066: HIF-1 signaling pathway	5	0.011951	TF, TFRC, INSR, AKT1, IGF1R
9	KEGG_PATHWAY	hsa04015: Rap1 signaling pathway	7	0.012957	ITGAM, ITGB3, INSR, ITGB2, AKT1, PRKCZ, IGF1R
10	KEGG_PATHWAY	hsa04114: oocyte meiosis	5	0.019438	CDC20, YWHAQ, FBXO5, SKP1, IGF1R
11	KEGG_PATHWAY	hsa03320: PPAR signaling pathway	4	0.024121	APOA2, APOC3, APOA1, PLTP
12	KEGG_PATHWAY	hsa04110: cell cycle	5	0.027856	CDC20, FZR1, SMAD3, YWHAQ, SKP1
13	KEGG_PATHWAY	hsa05166: HTLV-I infection	7	0.029982	CDC20, SMAD3, CANX, ITGB2, AKT1, CALR, ICAM1
14	KEGG_PATHWAY	hsa04640: hematopoietic cell lineage	4	0.046868	ITGAM, TFRC, ITGB3, CD22

**Table 6:** The significant enriched KEGG pathway analysis for module nodes.

	Category	Term	Count	*p* value	Genes
1	KEGG_PATHWAY	hsa03320: PPAR signaling pathway	3	0.001902	APOA2, APOC3, APOA1
2	KEGG_PATHWAY	hsa04610: complement and coagulation cascades	3	0.002016	FGB, FGA, FGG
3	KEGG_PATHWAY	hsa04611: platelet activation	3	0.006994	FGB, FGA, FGG
4	KEGG_PATHWAY	hsa04977: vitamin digestion and absorption	2	0.022183	APOA1, APOB
5	KEGG_PATHWAY	hsa04975: fat digestion and absorption	2	0.039034	APOA1, APOB

## Data Availability

All data generated or analyzed during this study are included in this published article (and its supplementary information files).

## Abbreviations

ITP: Idiopathic Thrombocytopenic Purpura
MF: Molecular Function
BP: Biological Process
CC: Cellular Component
GO: Gene Ontology
HP: Haptoglobin
KRT1: Keratin, type II cytoskeletal 1
APOA1: Apolipoprotein A-1
FGB: Fibrinogen beta chain
NTRK1: Neurotrophic receptor tyrosine kinase 1
CUL3: Cullin 3
HSPA8: Heat shock protein family A (Hsp70) member 8
HSPA5: Heat shock protein family A (Hsp70) member 5
HNRNPA1: Heterogeneous nuclear ribonucleoprotein A1
SERPINA1: Serpin family A member 1
FN1: Fibronectin 1
VCP: Valosin-containing protein
APP: Amyloid beta precursor protein
COPS5: COP9 signalosome subunit 5
KRT9: Keratin 9
VTDB: Vitamin D-binding protein
TF: Transferrin

## Additional Points

*Clinical Relevance*. The most prevalent autoimmune blood disorder is idiopathic thrombocytopenic purpura (ITP). A significant problem is the lack of biomarkers that can predict therapy responses. Following up on the drug therapy responses of the chronic ITP patients showed that they were divided into respondent and nonrespondent groups. Bioinformatic analysis based on the network helps to select crucial proteins from a wide range of proteins detected in proteomic analysis. Based on potential predictive biomarkers, APOA1, GC, and TF could categorize chronic ITP patients for drug therapy response.

## Supplementary Materials

Supplementary 1 Supplementary Tables: Supplementary Table S1: ITP patients information. Supplementary Table S2: data represented by Progenesis SameSpots software for nineteen differentially expressed protein spots (2-DE image analysis). Supplementary Table S3: the list of hubs and bottlenecks in networks, which is constructed based on HPRD for sixteen differentially expressed proteins. Supplementary Table S4: the enriched GO analysis of sixteen differentially expressed proteins based on significant *p* value. Supplementary Table S5: Gene Ontology enrichment based on biological process term for merged network nodes based on significant *p* value. Supplementary Table S6: Gene Ontology enrichment based on cellular component term for merged network nodes based on significant *p* value. Supplementary Table S7: Gene Ontology enrichment based on molecular function term for merged network nodes based on significant *p* value. Supplementary Table S8: the enriched GO analysis of the most important module of the PPI network based on significant *p* value.

Supplementary 2 Supplementary figures: Supplementary Figure S1: nineteen differentially expressed protein spots that were identified by Progenesis SameSpots software (2-DE image analysis). Supplementary Figure S2: 2-DE gel image of the control group. Supplementary Figure S3: The spectra of nineteen proteins, which are identified by MALDI‐TOF MS/MS. Supplementary Figure S4: this shows a graphical representation of the Mascot search results for nineteen identified proteins. Peptide mass fingerprints and MS/MS analysis were searched by the Mascot search engine against the SwissProt protein database. The parameters were utilized in the Mascot search engine: trypsin digestion: maximum one missed cleavage; fixed modification: carbamidomethylation (C); peptide mass tolerance: 1.2 Da; and mass value: [M+H]^+^ and monoisotopic. When the peptide score was above the cutoff value, protein identification was accepted (*p* < 0.05). Supplementary Figure S5: protein-protein interaction network of 16 differentially expressed proteins in respondents and nonrespondents was retrieved from the STRING database source. The PPI network consists of 16 nodes and 24 edges. Proteins are represented by nodes in the network, and PPI is represented by edges.
