# Supplementary material for: Proteomic-Based Discovery of Predictive Biomarkers for Drug Therapy Response and Personalized Medicine in Chronic Immune Thrombocytopenia
Source: Biomed Res Int. 2023 Oct 31;2023:9573863. doi: 10.1155/2023/9573863 (PMC10630023; doi:10.1155/2023/9573863)
Supplement: Supplementary 1 — Supplementary Tables: Supplementary Table S1: ITP patients information. Supplementary Table S2: data represented by Progenesis SameSpots software for nineteen differentially expressed protein spots (2-DE image analysis). Supplementary Table S3: the list of hubs and bottlenecks in networks, which is constructed based on HPRD for sixteen differentially expressed proteins. Supplementary Table S4: the enriched GO analysis of sixteen differentially expressed proteins based on significant p value. Supplementary Table S5: Gene Ontology enrichment based on biological process term for merged network nodes based on significant p value. Supplementary Table S6: Gene Ontology enrichment based on cellular component term for merged network nodes based on significant p value. Supplementary Table S7: Gene Ontology enrichment based on molecular function term for merged network nodes based on significant p value. Supplementary Table S8: the enriched GO analysis of the most important module of the PPI network based on significant p value. [file 9573863.f1.docx]

**Proteomic-based discovery of predictive biomarkers for drug therapy response and personalized medicine in chronic immune thrombocytopenia**

**Supplementary Tables:**

**Supplementary Table S1:** ITP Patient information

| Patient no | gender | age | First platelet count (per microliter) | Patients' response to drug therapy | Last Treatment | Last platelet count (per microliter) | outcome |
| --- | --- | --- | --- | --- | --- | --- | --- |
| 1 | female | 25 | 73000 | positive | Low dose prednisolone with azathioprine | 153000 | -Good response -no treatment |
| 2 | male | 58 | 86000 | positive | eltrombopag | 193000 | -Good response  -no treatment |
| 3 | female | 50 | 55000 | negative | splenectomy | 48000 | -Partial response  -no treatment |
| 4 | male | 36 | 65000 | negative | splenectomy | 45000 | --Partial response  -no treatment |
| 5 | female | 60 | 35000 | negative | splenectomy | 37000 | --Partial response  -no treatment |
| 6 | female | 23 | 2000 | negative | splenectomy | 39000 | --Partial response  -no treatment |
| 7 | male | 65 | 87000 | positive | eltrombopag | 198000 | --Good response  -no treatment |
| 8 | male | 15 | 65000 | positive | Mycophenolate mofetil  Low dose pprenisoslone | 225000 | --Good response  -no treatment |
| 9 | female | 42 | 42000 | positive | Low dose prednisolone azathioprine | 119000 | --Good response  -no treatment |
| 10 | male | 58 | 40000 | negative | splenectomy | 42000 | -partial response  -no treatment |
| 11 | male | 72 | 53000 | positive | Mycophenolate mofetil  Low dose prednisoslone | 289000 | --Good response  -no treatment |
| 12 | female | 32 | 22000 | negative | splenectomy | 44000 | -partial response  -no treatment |
| 13 | male | 25 | 16000 | negative | splenectomy | 33000 | -partial response  -no treatment |
| 14 | male | 56 | 11300 | positive | Low dose prednisolone with azathioprine | 118000 | --Good response  -no treatment |
| 15 | male | 49 | 11200 | positive | Low dose prednisolone azathioprine  Mycophenolate mofetil | 109000 | --Good response  -no treatment |
| 16 | male | 30 | 25000 | negative | splenectomy | 32000 | -partial response  -no treatment |

**Supplementary Table S2:** data represented by Progenesis SameSpots software for nineteen differentially expressed protein spots (2-DE image analysis)

| **#** | **Anova (p)** | **Fold** | **pI** | **MW** | **Average Normalised Volumes** | |
| --- | --- | --- | --- | --- | --- | --- |
|  |  |  |  |  | **PITP** | **NITP** |
| 380 | 9.358e-007 | 1.6 | 8.85 | 192 | 6376.000 | 4057.539 |
| 191 | 2.127e-012 | 1.7 | 6.7 | 159 | 1.468e+004 | 8838.000 |
| 205 | 2.285e-011 | 1.7 | 5.98 | 147 | 8480.804 | 4972.000 |
| 162 | 1.085e-005 | 1.7 | 5.91 | 173 | 2579.967 | 1503.000 |
| 381 | 1.604e-005 | 1.7 | 8.91 | 192 | 8787.813 | 5106.000 |
| 204 | 2.191e-006 | 1.9 | 5.59 | 149 | 8548.339 | 4541.000 |
| 348 | 1.624e-006 | 2.0 | 8.47 | 54 | 5961.753 | 1.186e+004 |
| 166 | 7.289e-007 | 2.0 | 8.99 | 169 | 6393.799 | 3186.000 |
| 242 | 2.179e-006 | 2.1 | 6.31 | 17 | 847.548 | 403.000 |
| 368 | 1.223e-007 | 2.2 | 5.69 | 162 | 1513.277 | 681.000 |
| 195 | 9.575e-009 | 2.4 | 5.73 | 157 | 934.884 | 389.000 |
| 206 | 1.692e-007 | 2.6 | 6.21 | 147 | 8979.020 | 3487.000 |
| 190 | 4.880e-006 | 2.6 | 5.8 | 160 | 1045.920 | 404.000 |
| 225 | 4.362e-006 | 3.1 | 6.13 | 66 | 2.327e+004 | 7427.000 |
| 177 | 8.844e-008 | 3.7 | 9.13 | 166 | 1.178e+004 | 3177.000 |
| 158 | 3.214e-008 | 3.7 | 6.03 | 175 | 2026.635 | 543.000 |
| 243 | 7.385e-009 | 3.8 | 7.15 | 17 | 1.432e+004 | 3773.000 |
| 246 | 2.245e-007 | 4.1 | 7.99 | 13 | 8731.216 | 2115.000 |
| 244 | 4.363e-011 | 5.7 | 6.02 | 15 | 3643.108 | 634.000 |

**Supplementary** **Table S3:** The list of hub and bottlenecks in networks which is constructed based on HPRD databases for sixteen differentially expressed proteins.

| Gene name | degree | Betwennes centrality |
| --- | --- | --- |
| **Hubs** | | |
| APOA1 | 21 | 0.34910108 |
| TF | 13 | 0.41894887 |
| FGG | 9 | 0.12540379 |
| KRT1 | 9 | 0.15236292 |
| GC | 9 | 0.1947883 |
| **Bottlenecks** | | |
| FBXO5 | 4 | 1 |
| TF | 13 | 0.41894887 |
| APOA1 | 21 | 0.34910108 |
| APOB | 4 | 0.26063176 |
| CALR | 4 | 0.26047335 |
| GC | 9 | 0.1947883 |
| FGB | 7 | 0.1544305 |
| KRT1 | 9 | 0.15236292 |
| TUBB3 | 2 | 0.14071996 |
| FGG | 9 | 0.12540379 |
| **Hub-Bottleneck** | | |
| APOA1 | 21 | 0.34910108 |
| TF | 13 | 0.41894887 |
| GC | 9 | 0.1947883 |
| KRT1 | 9 | 0.15236292 |

**Supplementary Table S4:** The enriched GO analysis of sixteen differentially expressed proteins based on significant P-Value.

|  | Category | Term | Count | P-Value | Genes |
| --- | --- | --- | --- | --- | --- |
|  | **Biological Process** | | | | |
| 1 | GOTERM_BP_DIRECT | GO:0002576~platelet degranulation | 4 | 9.67E-05 | FGB, TF, FGG, APOA1 |
| 2 | GOTERM_BP_DIRECT | GO:0042730~fibrinolysis | 3 | 1.55E-04 | FGB, KRT1, FGG |
| 3 | GOTERM_BP_DIRECT | GO:1900026~positive regulation of substrate adhesion-dependent cell spreading | 3 | 3.64E-04 | FGB, FGG, APOA1 |
| 4 | GOTERM_BP_DIRECT | GO:0072378~blood coagulation, fibrin clot formation | 2 | 0.003569 | FGB, FGG |
| 5 | GOTERM_BP_DIRECT | GO:0051180~vitamin transport | 2 | 0.004459 | APOA1, GC |
| 6 | GOTERM_BP_DIRECT | GO:0090277~positive regulation of peptide hormone secretion | 2 | 0.007125 | FGB, FGG |
| 7 | GOTERM_BP_DIRECT | GO:0031639~plasminogen activation | 2 | 0.008013 | FGB, FGG |
| 8 | GOTERM_BP_DIRECT | GO:0034116~positive regulation of heterotypic cell-cell adhesion | 2 | 0.009785 | FGB, FGG |
| 9 | GOTERM_BP_DIRECT | GO:0051258~protein polymerization | 2 | 0.011555 | FGB, FGG |
| 10 | GOTERM_BP_DIRECT | GO:0043623~cellular protein complex assembly | 2 | 0.015966 | FGB, FGG |
| 11 | GOTERM_BP_DIRECT | GO:0045921~positive regulation of exocytosis | 2 | 0.018603 | FGB, FGG |
| 12 | GOTERM_BP_DIRECT | GO:2000352~negative regulation of endothelial cell apoptotic process | 2 | 0.024732 | FGB, FGG |
| 13 | GOTERM_BP_DIRECT | GO:0045907~positive regulation of vasoconstriction | 2 | 0.028218 | FGB, FGG |
| 14 | GOTERM_BP_DIRECT | GO:1902042~negative regulation of extrinsic apoptotic signaling pathway via death domain receptors | 2 | 0.029088 | FGB, FGG |
| 15 | GOTERM_BP_DIRECT | GO:0050714~positive regulation of protein secretion | 2 | 0.031693 | FGB, FGG |
| 16 | GOTERM_BP_DIRECT | GO:0001895~retina homeostasis | 2 | 0.035156 | TF, KRT1 |
| 17 | GOTERM_BP_DIRECT | GO:0070527~platelet aggregation | 2 | 0.03602 | FGB, FGG |
|  | **Cellular Component** | | | | |
| 1 | GOTERM_CC_DIRECT | GO:0072562~blood microparticle | 7 | 1.43E-09 | FGB, TF, KRT1, FGG, HP, APOA1, GC |
| 2 | GOTERM_CC_DIRECT | GO:0005615~extracellular space | 9 | 3.51E-06 | FGB, TF, RBP4, KRT1, FGG, HP, APOA1, KRT10, GC |
| 3 | GOTERM_CC_DIRECT | GO:0070062~extracellular exosome | 11 | 1.07E-05 | FGB, TF, RBP4, KRT27, KRT1, FGG, HP, APOA1, SCRIB, KRT10, GC |
| 4 | GOTERM_CC_DIRECT | GO:0005576~extracellular region | 7 | 0.00117 | FGB, TF, RBP4, FGG, HP, APOA1, GC |
| 5 | GOTERM_CC_DIRECT | GO:0005577~fibrinogen complex | 2 | 0.007385 | FGB, FGG |
| 6 | GOTERM_CC_DIRECT | GO:0009986~cell surface | 4 | 0.009116 | FGB, TF, FGG, APOA1 |
| 7 | GOTERM_CC_DIRECT | GO:0034774~secretory granule lumen | 2 | 0.009835 | TF, APOA1 |
| 8 | GOTERM_CC_DIRECT | GO:0031091~platelet alpha granule | 2 | 0.011466 | FGB, FGG |
| 9 | GOTERM_CC_DIRECT | GO:0071682~endocytic vesicle lumen | 2 | 0.013094 | HP, APOA1 |
| 10 | GOTERM_CC_DIRECT | GO:1903561~extracellular vesicle | 2 | 0.040389 | FGB, APOA1 |
| 11 | GOTERM_CC_DIRECT | GO:0030139~endocytic vesicle | 2 | 0.043553 | TF, APOA1 |
| 12 | GOTERM_CC_DIRECT | GO:0031093~platelet alpha granule lumen | 2 | 0.044343 | FGB, FGG |
|  | **Molecular Function** | | | | |
| 1 | GOTERM_MF_DIRECT | GO:0005198~structural molecule activity | 4 | 1.00E-03 | FGB, KRT27, KRT1, FGG |
| 2 | GOTERM_MF_DIRECT | GO:0005515~protein binding | 12 | 0.0401263 | FGB, DYRK4, TF, RBP4, SEH1L, KRT1, FGG, HP, ZFP69B, APOA1, SCRIB, FBXO5 |

**Supplementary Table S5:** Gene ontology enrichment based on biological process term for merged networks nodes based on significant P-Value.

|  | | Category | | Term | | Count | | P-Value | | Genes | |
| --- | --- | --- | --- | --- | --- | --- | --- | --- | --- | --- | --- |
| 1 | GO:0042157 | | ~lipoprotein metabolic process | | 12 | | 4.75E-16 | | ABCA1, SCARB1, CUBN, CETP, ALB, APOA2, APOC3, LCAT, APOA1, APOB, LPA, APOL1 | |  |
| 2 | GOTERM_BP_DIRECT | | GO:0006898~receptor-mediated endocytosis | | 15 | | 1.44E-11 | | CETP, SCARB1, CUBN, CD163, HP, HBB, APOA1, LRP2, HPR, VTN, TFR2, ALB, CALR, APOB, APOL1 | |  |
| 3 | GOTERM_BP_DIRECT | | GO:0034375~high-density lipoprotein particle remodeling | | 7 | | 2.94E-10 | | SCARB1, CETP, APOA2, APOC3, LCAT, APOA1, PLTP | |  |
| 4 | GOTERM_BP_DIRECT | | GO:0008203~cholesterol metabolic process | | 10 | | 4.82E-10 | | ABCA1, CUBN, CETP, PON1, APOA2, APOF, LCAT, APOA1, APOB, APOL1 | |  |
| 5 | GOTERM_BP_DIRECT | | GO:0043691~reverse cholesterol transport | | 7 | | 1.07E-09 | | ABCA1, SCARB1, CETP, APOA2, APOC3, LCAT, APOA1 | |  |
| 6 | GOTERM_BP_DIRECT | | GO:0006869~lipid transport | | 9 | | 2.96E-08 | | CETP, APOA2, APOF, APOC3, APOA1, LPA, APOL1, PLTP, SERPINA5 | |  |
| 7 | GOTERM_BP_DIRECT | | GO:0043567~regulation of insulin-like growth factor receptor signaling pathway | | 5 | | 5.58E-08 | | IGFBP1, IGFBP4, IGFBP3, IGFBP2, IGFBP6 | |  |
| 8 | GOTERM_BP_DIRECT | | GO:0001523~retinoid metabolic process | | 8 | | 1.23E-07 | | RBP4, TTR, APOA2, APOC3, APOA1, LRP2, APOB, RPE65 | |  |
| 9 | GOTERM_BP_DIRECT | | GO:0042632~cholesterol homeostasis | | 8 | | 1.72E-07 | | ABCA1, SCARB1, CETP, APOA2, APOC3, LCAT, APOA1, APOB | |  |
| 10 | GOTERM_BP_DIRECT | | GO:0002576~platelet degranulation | | 9 | | 3.23E-07 | | FGB, FGA, TF, ITGB3, ALB, FGG, F13A1, APOA1, KNG1 | |  |
| 11 | GOTERM_BP_DIRECT | | GO:0033344~cholesterol efflux | | 6 | | 4.81E-07 | | ABCA1, SCARB1, APOA2, APOC3, APOA1, APOB | |  |
| 12 | GOTERM_BP_DIRECT | | GO:0030198~extracellular matrix organization | | 11 | | 5.36E-07 | | FGB, FGA, VTN, ITGAM, TTR, ITGB3, ITGB2, FGG, FBLN1, NID1, ICAM1 | |  |
| 13 | GOTERM_BP_DIRECT | | GO:0044267~cellular protein metabolic process | | 9 | | 9.18E-07 | | IGFBP1, FGA, TTR, IGFBP5, IGFBP4, IGFBP3, IGFBP2, APOA1, IGFBP6 | |  |
| 14 | GOTERM_BP_DIRECT | | GO:0072378~blood coagulation, fibrin clot formation | | 4 | | 1.03E-06 | | FGB, FGA, FGG, FBLN1 | |  |
| 15 | GOTERM_BP_DIRECT | | GO:1900026~positive regulation of substrate adhesion-dependent cell spreading | | 6 | | 1.76E-06 | | FGB, FGA, FGG, APOA1, CALR, ARHGEF7 | |  |
| 16 | GOTERM_BP_DIRECT | | GO:0045429~positive regulation of nitric oxide biosynthetic process | | 6 | | 7.94E-06 | | SMAD3, INSR, ITGB2, HBB, AKT1, ICAM1 | |  |
| 17 | GOTERM_BP_DIRECT | | GO:0042730~fibrinolysis | | 5 | | 8.90E-06 | | FGB, FGA, F12, KRT1, FGG | |  |
| 18 | GOTERM_BP_DIRECT | | GO:0008286~insulin receptor signaling pathway | | 7 | | 1.09E-05 | | IGFBP1, INSR, AKT1, PRKCZ, GPLD1, RPE65, IGF1R | |  |
| 19 | GOTERM_BP_DIRECT | | GO:0001558~regulation of cell growth | | 7 | | 1.26E-05 | | IGFBP1, IGFBP5, TFRC, IGFBP4, IGFBP3, IGFBP2, IGFBP6 | |  |
| 20 | GOTERM_BP_DIRECT | | GO:0090277~positive regulation of peptide hormone secretion | | 4 | | 1.42E-05 | | FGB, FGA, TFR2, FGG | |  |
| 21 | GOTERM_BP_DIRECT | | GO:0042158~lipoprotein biosynthetic process | | 4 | | 2.11E-05 | | APOA2, LCAT, APOA1, APOB | |  |
| 22 | GOTERM_BP_DIRECT | | GO:0007160~cell-matrix adhesion | | 7 | | 2.47E-05 | | FGB, FGA, VTN, ITGB3, ITGB2, FGG, NID1 | |  |
| 23 | GOTERM_BP_DIRECT | | GO:0046470~phosphatidylcholine metabolic process | | 4 | | 3.00E-05 | | CETP, PON1, LCAT, GPLD1 | |  |
| 24 | GOTERM_BP_DIRECT | | GO:0045907~positive regulation of vasoconstriction | | 5 | | 5.07E-05 | | FGB, FGA, FGG, AKT1, ICAM1 | |  |
| 25 | GOTERM_BP_DIRECT | | GO:0051258~protein polymerization | | 4 | | 7.06E-05 | | FGB, FGA, VTN, FGG | |  |
| 26 | GOTERM_BP_DIRECT | | GO:0033700~phospholipid efflux | | 4 | | 8.94E-05 | | ABCA1, APOA2, APOC3, APOA1 | |  |
| 27 | GOTERM_BP_DIRECT | | GO:0006953~acute-phase response | | 5 | | 1.12E-04 | | CD163, TFR2, HP, LBP, MBL2 | |  |
| 28 | GOTERM_BP_DIRECT | | GO:0010903~negative regulation of very-low-density lipoprotein particle remodeling | | 3 | | 1.22E-04 | | APOA2, APOC3, APOA1 | |  |
| 29 | GOTERM_BP_DIRECT | | GO:0030301~cholesterol transport | | 4 | | 1.36E-04 | | CETP, LCAT, APOA1, APOB | |  |
| 30 | GOTERM_BP_DIRECT | | GO:0070527~platelet aggregation | | 5 | | 1.36E-04 | | FGB, FGA, ITGB3, FGG, HBB | |  |
| 31 | GOTERM_BP_DIRECT | | GO:0007596~blood coagulation | | 8 | | 1.86E-04 | | FGB, FGA, ITGB3, FGG, F13A1, HBB, F13B, SERPINA5 | |  |
| 32 | GOTERM_BP_DIRECT | | GO:0090131~mesenchyme migration | | 3 | | 4.05E-04 | | ACTA2, ACTA1, ACTC1 | |  |
| 33 | GOTERM_BP_DIRECT | | GO:0032489~regulation of Cdc42 protein signal transduction | | 3 | | 4.05E-04 | | ABCA1, APOC3, APOA1 | |  |
| 34 | GOTERM_BP_DIRECT | | GO:0040020~regulation of meiotic nuclear division | | 3 | | 4.05E-04 | | CDC20, FZR1, CALR | |  |
| 35 | GOTERM_BP_DIRECT | | GO:0034198~cellular response to amino acid starvation | | 4 | | 5.37E-04 | | SEH1L, MIOS, NPRL2, WDR24 | |  |
| 36 | GOTERM_BP_DIRECT | | GO:0070328~triglyceride homeostasis | | 4 | | 6.04E-04 | | SCARB1, CETP, APOC3, APOA1 | |  |
| 37 | GOTERM_BP_DIRECT | | GO:0034384~high-density lipoprotein particle clearance | | 3 | | 6.05E-04 | | SCARB1, APOA2, APOA1 | |  |
| 38 | GOTERM_BP_DIRECT | | GO:2000352~negative regulation of endothelial cell apoptotic process | | 4 | | 7.54E-04 | | FGB, FGA, FGG, ICAM1 | |  |
| 39 | GOTERM_BP_DIRECT | | GO:0030168~platelet activation | | 6 | | 8.78E-04 | | FGB, FGA, ITGB3, PRKCE, FGG, AKT1 | |  |
| 40 | GOTERM_BP_DIRECT | | GO:0010951~negative regulation of endopeptidase activity | | 6 | | 0.001103 | | C3, VTN, AKT1, LPA, KNG1, SERPINA5 | |  |
| 41 | GOTERM_BP_DIRECT | | GO:0034380~high-density lipoprotein particle assembly | | 3 | | 0.001119 | | ABCA1, APOA2, APOA1 | |  |
| 42 | GOTERM_BP_DIRECT | | GO:0031663~lipopolysaccharide-mediated signaling pathway | | 4 | | 0.00112 | | SCARB1, PRKCE, AKT1, LBP | |  |
| 43 | GOTERM_BP_DIRECT | | GO:1902042~negative regulation of extrinsic apoptotic signaling pathway via death domain receptors | | 4 | | 0.001227 | | FGB, FGA, FGG, ICAM1 | |  |
| 44 | GOTERM_BP_DIRECT | | GO:0055091~phospholipid homeostasis | | 3 | | 0.001433 | | ABCA1, CETP, APOA1 | |  |
| 45 | GOTERM_BP_DIRECT | | GO:0031639~plasminogen activation | | 3 | | 0.001433 | | FGB, FGA, FGG | |  |
| 46 | GOTERM_BP_DIRECT | | GO:0044342~type B pancreatic cell proliferation | | 3 | | 0.001433 | | IGFBP5, IGFBP4, IGFBP3 | |  |
| 47 | GOTERM_BP_DIRECT | | GO:0042060~wound healing | | 5 | | 0.001759 | | DSP, SCARB1, SMAD3, ITGB3, SCRIB | |  |
| 48 | GOTERM_BP_DIRECT | | GO:0042359~vitamin D metabolic process | | 3 | | 0.001783 | | CUBN, LRP2, GC | |  |
| 49 | GOTERM_BP_DIRECT | | GO:0001895~retina homeostasis | | 4 | | 0.00215 | | TF, ALB, KRT1, RPE65 | |  |
| 50 | GOTERM_BP_DIRECT | | GO:0034116~positive regulation of heterotypic cell-cell adhesion | | 3 | | 0.00217 | | FGB, FGA, FGG | |  |
| 51 | GOTERM_BP_DIRECT | | GO:0034374~low-density lipoprotein particle remodeling | | 3 | | 0.00217 | | CETP, APOA2, APOB | |  |
| 52 | GOTERM_BP_DIRECT | | GO:0010763~positive regulation of fibroblast migration | | 3 | | 0.00217 | | PRKCE, AKT1, ARHGEF7 | |  |
| 53 | GOTERM_BP_DIRECT | | GO:0007165~signal transduction | | 17 | | 0.003007 | | FGB, IGFBP1, FGA, IGFBP5, IGFBP4, PRKCE, PDE1A, FGG, IGFBP2, TNFRSF10C, PRKCZ, IGF1R, C3, SH3BP2, AKT1, IGFBP6, ARHGEF7 | |  |
| 54 | GOTERM_BP_DIRECT | | GO:0010745~negative regulation of macrophage derived foam cell differentiation | | 3 | | 0.003052 | | ABCA1, CETP, ITGB3 | |  |
| 55 | GOTERM_BP_DIRECT | | GO:0043568~positive regulation of insulin-like growth factor receptor signaling pathway | | 3 | | 0.003052 | | IGFBP5, IGFBP4, IGFBP3 | |  |
| 56 | GOTERM_BP_DIRECT | | GO:0006629~lipid metabolic process | | 6 | | 0.00345 | | ATP5B, APOF, LCAT, LRP2, LPA, PLTP | |  |
| 57 | GOTERM_BP_DIRECT | | GO:0010875~positive regulation of cholesterol efflux | | 3 | | 0.003546 | | ABCA1, PON1, PLTP | |  |
| 58 | GOTERM_BP_DIRECT | | GO:0007229~integrin-mediated signaling pathway | | 5 | | 0.003814 | | ITGAM, ITGB3, ITGB2, APOA1, FBLN1 | |  |
| 59 | GOTERM_BP_DIRECT | | GO:0033627~cell adhesion mediated by integrin | | 3 | | 0.004075 | | VTN, ITGB3, ICAM1 | |  |
| 60 | GOTERM_BP_DIRECT | | GO:0042953~lipoprotein transport | | 3 | | 0.004075 | | CUBN, LRP2, APOB | |  |
| 61 | GOTERM_BP_DIRECT | | GO:0007568~aging | | 6 | | 0.004261 | | IGFBP1, IGFBP5, CANX, ITGB2, IGFBP2, AKT1 | |  |
| 62 | GOTERM_BP_DIRECT | | GO:0043623~cellular protein complex assembly | | 3 | | 0.005863 | | FGB, FGA, FGG | |  |
| 63 | GOTERM_BP_DIRECT | | GO:0034142~toll-like receptor 4 signaling pathway | | 3 | | 0.005863 | | ITGAM, ITGB2, LBP | |  |
| 64 | GOTERM_BP_DIRECT | | GO:0010628~positive regulation of gene expression | | 7 | | 0.006933 | | ACTA2, ACTA1, ACTC1, SMAD3, FBLN1, CALR, APOB | |  |
| 65 | GOTERM_BP_DIRECT | | GO:0045921~positive regulation of exocytosis | | 3 | | 0.007947 | | FGB, FGA, FGG | |  |
| 66 | GOTERM_BP_DIRECT | | GO:0050900~leukocyte migration | | 5 | | 0.007955 | | ITGAM, ITGB3, ITGB2, APOB, ICAM1 | |  |
| 67 | GOTERM_BP_DIRECT | | GO:0010906~regulation of glucose metabolic process | | 3 | | 0.008705 | | IGFBP5, IGFBP4, IGFBP3 | |  |
| 68 | GOTERM_BP_DIRECT | | GO:0051439~regulation of ubiquitin-protein ligase activity involved in mitotic cell cycle | | 3 | | 0.009494 | | CDC20, FZR1, FBXO5 | |  |
| 69 | GOTERM_BP_DIRECT | | GO:0006656~phosphatidylcholine biosynthetic process | | 3 | | 0.011165 | | APOA2, LCAT, APOA1 | |  |
| 70 | GOTERM_BP_DIRECT | | GO:0019433~triglyceride catabolic process | | 3 | | 0.011165 | | APOC3, APOA1, APOB | |  |
| 71 | GOTERM_BP_DIRECT | | GO:0016032~viral process | | 7 | | 0.012768 | | NUP107, SEH1L, NUP85, APOA2, KRT7, FBLN1, SCRIB | |  |
| 72 | GOTERM_BP_DIRECT | | GO:0090107~regulation of high-density lipoprotein particle assembly | | 2 | | 0.012822 | | ABCA1, LCAT | |  |
| 73 | GOTERM_BP_DIRECT | | GO:0060621~negative regulation of cholesterol import | | 2 | | 0.012822 | | APOA2, APOC3 | |  |
| 74 | GOTERM_BP_DIRECT | | GO:0097460~ferrous iron import into cell | | 2 | | 0.012822 | | TF, TFR2 | |  |
| 75 | GOTERM_BP_DIRECT | | GO:0015920~lipopolysaccharide transport | | 2 | | 0.012822 | | SCARB1, LBP | |  |
| 76 | GOTERM_BP_DIRECT | | GO:0051437~positive regulation of ubiquitin-protein ligase activity involved in regulation of mitotic cell cycle transition | | 4 | | 0.01295 | | CDC20, FZR1, FBXO5, SKP1 | |  |
| 77 | GOTERM_BP_DIRECT | | GO:0032008~positive regulation of TOR signaling | | 3 | | 0.012954 | | SEH1L, MIOS, WDR24 | |  |
| 78 | GOTERM_BP_DIRECT | | GO:0042493~response to drug | | 7 | | 0.01376 | | ABCA1, ACTC1, IGFBP2, APOA2, APOA1, CALR, ICAM1 | |  |
| 79 | GOTERM_BP_DIRECT | | GO:0046718~viral entry into host cell | | 4 | | 0.014859 | | SCARB1, TFRC, ITGB3, ICAM1 | |  |
| 80 | GOTERM_BP_DIRECT | | GO:0050766~positive regulation of phagocytosis | | 3 | | 0.01486 | | C3, CALR, MBL2 | |  |
| 81 | GOTERM_BP_DIRECT | | GO:0043410~positive regulation of MAPK cascade | | 4 | | 0.015359 | | IGFBP4, INSR, PRKCE, IGFBP3 | |  |
| 82 | GOTERM_BP_DIRECT | | GO:0042572~retinol metabolic process | | 3 | | 0.015855 | | RBP4, TTR, RPE65 | |  |
| 83 | GOTERM_BP_DIRECT | | GO:0046326~positive regulation of glucose import | | 3 | | 0.015855 | | INSR, AKT1, PRKCZ | |  |
| 84 | GOTERM_BP_DIRECT | | GO:0008544~epidermis development | | 4 | | 0.017457 | | DSP, INSR, KRT5, EVPL | |  |
| 85 | GOTERM_BP_DIRECT | | GO:0006409~tRNA export from nucleus | | 3 | | 0.017929 | | NUP107, SEH1L, NUP85 | |  |
| 86 | GOTERM_BP_DIRECT | | GO:0010827~regulation of glucose transport | | 3 | | 0.019006 | | NUP107, SEH1L, NUP85 | |  |
| 89 | GOTERM_BP_DIRECT | | GO:0018158~protein oxidation | | 2 | | 0.019172 | | APOA2, APOA1 | |  |
| 90 | GOTERM_BP_DIRECT | | GO:0044406~adhesion of symbiont to host | | 2 | | 0.019172 | | SCARB1, ICAM1 | |  |
| 91 | GOTERM_BP_DIRECT | | GO:0033572~transferrin transport | | 3 | | 0.021241 | | TF, TFRC, TFR2 | |  |
| 92 | GOTERM_BP_DIRECT | | GO:0006641~triglyceride metabolic process | | 3 | | 0.021241 | | CETP, APOA2, APOC3 | |  |
| 93 | GOTERM_BP_DIRECT | | GO:0050714~positive regulation of protein secretion | | 3 | | 0.022397 | | FGB, FGA, FGG | |  |
| 94 | GOTERM_BP_DIRECT | | GO:0010811~positive regulation of cell-substrate adhesion | | 3 | | 0.024786 | | VTN, PRKCE, NID1 | |  |
| 95 | GOTERM_BP_DIRECT | | GO:0010897~negative regulation of triglyceride catabolic process | | 2 | | 0.025482 | | APOC3, GPLD1 | |  |
| 96 | GOTERM_BP_DIRECT | | GO:0043152~induction of bacterial agglutination | | 2 | | 0.025482 | | FGB, FGA | |  |
| 97 | GOTERM_BP_DIRECT | | GO:0060192~negative regulation of lipase activity | | 2 | | 0.025482 | | APOA2, APOA1 | |  |
| 98 | GOTERM_BP_DIRECT | | GO:0071896~protein localization to adherens junction | | 2 | | 0.025482 | | DSP, SCRIB | |  |
| 99 | GOTERM_BP_DIRECT | | GO:0051301~cell division | | 7 | | 0.025528 | | CDC20, FZR1, SEH1L, PRKCE, FBXO5, EVI5, CDC73 | |  |
| 100 | GOTERM_BP_DIRECT | | GO:0070374~positive regulation of ERK1 and ERK2 cascade | | 5 | | 0.026454 | | FGB, FGA, FGG, PRKCZ, ICAM1 | |  |
| 101 | GOTERM_BP_DIRECT | | GO:0042593~glucose homeostasis | | 4 | | 0.027372 | | RBP4, IGFBP5, INSR, AKT1 | |  |
| 102 | GOTERM_BP_DIRECT | | GO:0007155~cell adhesion | | 8 | | 0.02869 | | VTN, ITGAM, ITGB3, PRKCE, ITGB2, LPP, CD22, ICAM1 | |  |
| 103 | GOTERM_BP_DIRECT | | GO:0007062~sister chromatid cohesion | | 4 | | 0.028783 | | CDC20, NUP107, SEH1L, NUP85 | |  |
| 104 | GOTERM_BP_DIRECT | | GO:0001649~osteoblast differentiation | | 4 | | 0.029503 | | ATP5B, IGFBP5, IGFBP3, AKT1 | |  |
| 105 | GOTERM_BP_DIRECT | | GO:0045740~positive regulation of DNA replication | | 3 | | 0.029858 | | INSR, CALR, IGF1R | |  |
| 106 | GOTERM_BP_DIRECT | | GO:0030335~positive regulation of cell migration | | 5 | | 0.031014 | | SMAD3, INSR, TRIP6, STX4, IGF1R | |  |
| 107 | GOTERM_BP_DIRECT | | GO:0038027~apolipoprotein A-I-mediated signaling pathway | | 2 | | 0.031751 | | ABCA1, ITGB3 | |  |
| 108 | GOTERM_BP_DIRECT | | GO:0030240~skeletal muscle thin filament assembly | | 2 | | 0.031751 | | ACTA1, ACTC1 | |  |
| 109 | GOTERM_BP_DIRECT | | GO:0051180~vitamin transport | | 2 | | 0.031751 | | APOA1, GC | |  |
| 110 | GOTERM_BP_DIRECT | | GO:0050996~positive regulation of lipid catabolic process | | 2 | | 0.031751 | | PRKCE, APOA2 | |  |
| 111 | GOTERM_BP_DIRECT | | GO:0030300~regulation of intestinal cholesterol absorption | | 2 | | 0.031751 | | APOA2, APOA1 | |  |
| 112 | GOTERM_BP_DIRECT | | GO:0007077~mitotic nuclear envelope disassembly | | 3 | | 0.032536 | | NUP107, SEH1L, NUP85 | |  |
| 113 | GOTERM_BP_DIRECT | | GO:0006879~cellular iron ion homeostasis | | 3 | | 0.032536 | | TF, TFRC, TFR2 | |  |
| 114 | GOTERM_BP_DIRECT | | GO:0010595~positive regulation of endothelial cell migration | | 3 | | 0.035304 | | SCARB1, ITGB3, GPLD1 | |  |
| 115 | GOTERM_BP_DIRECT | | GO:0006954~inflammatory response | | 7 | | 0.035652 | | C3, IGFBP4, ITGB2, TNFRSF10C, AKT1, PRKCZ, KNG1 | |  |
| 116 | GOTERM_BP_DIRECT | | GO:0071222~cellular response to lipopolysaccharide | | 4 | | 0.036408 | | ABCA1, LBP, CDC73, ICAM1 | |  |
| 117 | GOTERM_BP_DIRECT | | GO:0018206~peptidyl-methionine modification | | 2 | | 0.03798 | | APOA2, APOA1 | |  |
| 118 | GOTERM_BP_DIRECT | | GO:0070508~cholesterol import | | 2 | | 0.03798 | | SCARB1, APOA1 | |  |
| 119 | GOTERM_BP_DIRECT | | GO:0002740~negative regulation of cytokine secretion involved in immune response | | 2 | | 0.03798 | | APOA2, APOA1 | |  |
| 120 | GOTERM_BP_DIRECT | | GO:0010694~positive regulation of alkaline phosphatase activity | | 2 | | 0.03798 | | SMAD3, GPLD1 | |  |
| 121 | GOTERM_BP_DIRECT | | GO:0071281~cellular response to iron ion | | 2 | | 0.03798 | | TF, TFR2 | |  |
| 122 | GOTERM_BP_DIRECT | | GO:0051346~negative regulation of hydrolase activity | | 2 | | 0.03798 | | PRKCZ, SERPINA5 | |  |
| 123 | GOTERM_BP_DIRECT | | GO:0018149~peptide cross-linking | | 3 | | 0.041102 | | DSP, F13A1, EVPL | |  |
| 124 | GOTERM_BP_DIRECT | | GO:0075733~intracellular transport of virus | | 3 | | 0.042604 | | NUP107, SEH1L, NUP85 | |  |
| 125 | GOTERM_BP_DIRECT | | GO:0034372~very-low-density lipoprotein particle remodeling | | 2 | | 0.04417 | | CETP, LCAT | |  |
| 126 | GOTERM_BP_DIRECT | | GO:0010886~positive regulation of cholesterol storage | | 2 | | 0.04417 | | SCARB1, APOB | |  |
| 127 | GOTERM_BP_DIRECT | | GO:0001867~complement activation, lectin pathway | | 2 | | 0.04417 | | KRT1, MBL2 | |  |
| 128 | GOTERM_BP_DIRECT | | GO:0043065~positive regulation of apoptotic process | | 6 | | 0.044399 | | IGFBP3, AKT1, SCRIB, ARHGEF7, KNG1, GPLD1 | |  |
| 129 | GOTERM_BP_DIRECT | | GO:0001934~positive regulation of protein phosphorylation | | 4 | | 0.048645 | | C3, ITGB3, INSR, AKT1 | |  |

**Supplementary Table S6:** Gene ontology enrichment based on cellular components term for merged networks nodes based on significant P-Value.

|  | Category | Term | Count | P-Value | Genes |
| --- | --- | --- | --- | --- | --- |
| 1 | GOTERM_CC_DIRECT | GO:0070062~extracellular exosome | 63 | 1.05E-23 | SCARB1, ITGAM, TFRC, ITGB3, ITGB2, HP, HBB, ICAM1, SERPINA5, PCMT1, YWHAQ, TUBB3, KRT27, C1RL, LBP, SKP1, DSP, FGB, FGA, CUBN, IGFBP3, KRT1, IGFBP2, FGG, APOA2, KRT7, APOA1, SCRIB, KRT5, ACTA2, ACTA1, RBP4, CANX, GPLD1, CETP, NAXE, PON1, LCAT, FBLN1, HPR, LRP2, NID1, EVPL, PRKCZ, KNG1, C3, VTN, ATP5B, TTR, STX4, IGFBP6, GC, APOB, INSR, F12, APOC3, KRT10, TF, ACTC1, CFHR1, ALB, CALR, CD22 |
| 2 | GOTERM_CC_DIRECT | GO:0072562~blood microparticle | 22 | 3.45E-23 | FGB, FGA, TFRC, PON1, KRT1, FGG, HP, APOA2, HBB, APOA1, F13A1, HPR, KNG1, C3, VTN, ACTA1, TF, ACTC1, CFHR1, ALB, GC, APOL1 |
| 3 | GOTERM_CC_DIRECT | GO:0005615~extracellular space | 45 | 2.89E-22 | CETP, ITGAM, NAXE, TFRC, PON1, HP, LCAT, FBLN1, KNG1, SERPINA5, ICAM1, C3, ADAMTS4, VTN, TTR, C1RL, STX4, LBP, IGFBP6, GC, APOB, APOL1, PLTP, MBL2, FGB, IGFBP1, FGA, IGFBP4, IGFBP3, F12, KRT1, FGG, IGFBP2, APOA1, APOC3, KRT10, ACTA2, ACTA1, TF, RBP4, ACTC1, CFHR1, ALB, CALR, GPLD1 |
| 4 | GOTERM_CC_DIRECT | GO:0005576~extracellular region | 44 | 2.17E-18 | CETP, NAXE, TFRC, PON1, HP, LCAT, HBB, F13A1, FBLN1, HPR, NID1, KNG1, SERPINA5, C3, ADAMTS4, VTN, TTR, APOF, LBP, IGFBP6, GC, APOB, APOL1, PLTP, MBL2, FGB, IGFBP1, FGA, CD163, IGFBP5, IGFBP4, IGFBP3, F12, FGG, IGFBP2, APOA2, APOA1, APOC3, TF, RBP4, ALB, F13B, CALR, LPA |
| 5 | GOTERM_CC_DIRECT | GO:0034364~high-density lipoprotein particle | 8 | 3.34E-11 | ABCA1, CETP, PON1, APOA2, APOF, LCAT, APOA1, APOL1 |
| 6 | GOTERM_CC_DIRECT | GO:0034366~spherical high-density lipoprotein particle | 5 | 8.02E-08 | PON1, APOA2, APOC3, APOA1, HPR |
| 7 | GOTERM_CC_DIRECT | GO:0009986~cell surface | 16 | 7.13E-07 | FGB, FGA, SCARB1, ITGAM, TFRC, ITGB3, ITGB2, FGG, APOA1, ICAM1, ATP5B, TF, STX4, CALR, LBP, MBL2 |
| 8 | GOTERM_CC_DIRECT | GO:0071682~endocytic vesicle lumen | 5 | 2.01E-06 | HP, HBB, APOA1, CALR, APOB |
| 9 | GOTERM_CC_DIRECT | GO:0034361~very-low-density lipoprotein particle | 5 | 5.25E-06 | APOA2, APOC3, APOA1, APOB, APOL1 |
| 10 | GOTERM_CC_DIRECT | GO:1903561~extracellular vesicle | 6 | 1.14E-05 | FGB, FGA, PCMT1, TFRC, ITGB2, APOA1 |
| 11 | GOTERM_CC_DIRECT | GO:0005577~fibrinogen complex | 4 | 1.66E-05 | FGB, FGA, FGG, FBLN1 |
| 12 | GOTERM_CC_DIRECT | GO:0031093~platelet alpha granule lumen | 6 | 1.83E-05 | FGB, FGA, ALB, FGG, F13A1, KNG1 |
| 13 | GOTERM_CC_DIRECT | GO:0009897~external side of plasma membrane | 9 | 3.95E-05 | ABCA1, FGB, FGA, TFRC, TFR2, FGG, CALR, SERPINA5, ICAM1 |
| 14 | GOTERM_CC_DIRECT | GO:0031012~extracellular matrix | 10 | 6.68E-05 | ADAMTS4, DSP, VTN, ATP5B, CANX, KRT1, FBLN1, CALR, NID1, TOMM20 |
| 15 | GOTERM_CC_DIRECT | GO:0042627~chylomicron | 4 | 7.03E-05 | APOA2, APOC3, APOA1, APOB |
| 16 | GOTERM_CC_DIRECT | GO:0031091~platelet alpha granule | 4 | 7.03E-05 | FGB, FGA, FGG, SERPINA5 |
| 17 | GOTERM_CC_DIRECT | GO:0043235~receptor complex | 7 | 1.09E-04 | SMAD3, ITGB3, INSR, ITGB2, INSRR, LRP2, IGF1R |
| 18 | GOTERM_CC_DIRECT | GO:0030139~endocytic vesicle | 5 | 2.94E-04 | ABCA1, CUBN, TF, APOA1, LRP2 |
| 19 | GOTERM_CC_DIRECT | GO:0044297~cell body | 5 | 5.31E-04 | ACTA2, ACTA1, ACTC1, NAXE, RPE65 |
| 20 | GOTERM_CC_DIRECT | GO:0005819~spindle | 6 | 7.68E-04 | CDC20, NUP85, MAP7D1, AKT1, FBXO5, EVI5 |
| 21 | GOTERM_CC_DIRECT | GO:0043234~protein complex | 10 | 7.69E-04 | ACTA2, CDC20, RBP4, TTR, YWHAQ, CANX, ALB, AKT1, ARHGEF7, SERPINA5 |
| 22 | GOTERM_CC_DIRECT | GO:1990712~HFE-transferrin receptor complex | 3 | 9.52E-04 | TF, TFRC, TFR2 |
| 23 | GOTERM_CC_DIRECT | GO:0031080~nuclear pore outer ring | 3 | 0.001518 | NUP107, SEH1L, NUP85 |
| 24 | GOTERM_CC_DIRECT | GO:0061700~GATOR2 complex | 3 | 0.001518 | SEH1L, MIOS, WDR24 |
| 25 | GOTERM_CC_DIRECT | GO:0005829~cytosol | 33 | 0.0024968 | SEH1L, NUP107, NAXE, PDE1A, HBB, PRKCZ, CALB2, CDC20, PCMT1, FZR1, YWHAQ, NUP85, AKT1, STX4, FBXO5, GC, APOB, EVI5, SERF2, SKP1, CUBN, SMAD3, PRKCE, APOA2, APOA1, KRT5, ACTA2, ACTA1, RBP4, ACTC1, CALR, ARHGEF7, TJP2 |
| 26 | GOTERM_CC_DIRECT | GO:0030027~lamellipodium | 6 | 0.0026415 | ACTA2, ACTA1, ACTC1, SCRIB, STX4, ARHGEF7 |
| 27 | GOTERM_CC_DIRECT | GO:0005905~clathrin-coated pit | 4 | 0.0042457 | CUBN, TF, TFRC, LRP2 |
| 28 | GOTERM_CC_DIRECT | GO:0016020~membrane | 24 | 0.0045042 | CUBN, CD163, NUP107, TFRC, INSR, KRT1, ITGB2, INSRR, KRT5, KRT10, PRKCZ, RPE65, SERPINA5, ICAM1, IGF1R, ATP5B, ACTC1, EXOSC10, YWHAQ, NUP85, CANX, STX4, CALR, LBP |
| 29 | GOTERM_CC_DIRECT | GO:0005882~intermediate filament | 5 | 0.0045795 | DSP, KRT27, KRT7, KRT10, KRT5 |
| 30 | GOTERM_CC_DIRECT | GO:0043231~intracellular membrane-bounded organelle | 10 | 0.0059266 | SCARB1, DYRK4, NAXE, TFRC, INSR, PON1, APOB, EVI5, GPLD1, IGF1R |
| 31 | GOTERM_CC_DIRECT | GO:0005886~plasma membrane | 37 | 0.0068855 | SCARB1, ITGAM, TFRC, ITGB3, ITGB2, LRP2, PRKCZ, LPP, KNG1, IGF1R, ICAM1, C3, ATP5B, AKT1, STX4, APOB, FGB, ABCA1, DSP, FGA, CUBN, CD163, SMAD3, PRKCE, F12, INSR, KRT1, FGG, TNFRSF10C, APOA1, SCRIB, KRT5, RPE65, TRIP6, ARHGEF7, CD22, TJP2 |
| 32 | GOTERM_CC_DIRECT | GO:0005925~focal adhesion | 8 | 0.0086765 | ACTC1, YWHAQ, ITGB3, TRIP6, CALR, ARHGEF7, LPP, ICAM1 |
| 33 | GOTERM_CC_DIRECT | GO:0008305~integrin complex | 3 | 0.011089 | ITGAM, ITGB3, ITGB2 |
| 34 | GOTERM_CC_DIRECT | GO:0000322~storage vacuole | 2 | 0.0176746 | STX4, ARHGEF7 |
| 35 | GOTERM_CC_DIRECT | GO:0016942~insulin-like growth factor binding protein complex | 2 | 0.0176746 | IGFBP5, IGFBP3 |
| 36 | GOTERM_CC_DIRECT | GO:0071062~alphav-beta3 integrin-vitronectin complex | 2 | 0.0176746 | VTN, ITGB3 |
| 37 | GOTERM_CC_DIRECT | GO:0071944~cell periphery | 3 | 0.022358 | TUBB3, PRKCE, NID1 |
| 38 | GOTERM_CC_DIRECT | GO:0031838~haptoglobin-hemoglobin complex | 2 | 0.023497 | HP, HBB |
| 39 | GOTERM_CC_DIRECT | GO:0042567~insulin-like growth factor ternary complex | 2 | 0.023497 | IGFBP5, IGFBP3 |
| 40 | GOTERM_CC_DIRECT | GO:0035867~alphav-beta3 integrin-IGF-1-IGF1R complex | 2 | 0.023497 | ITGB3, IGF1R |
| 41 | GOTERM_CC_DIRECT | GO:0034363~intermediate-density lipoprotein particle | 2 | 0.023497 | APOC3, APOB |
| 42 | GOTERM_CC_DIRECT | GO:0005788~endoplasmic reticulum lumen | 5 | 0.0274239 | CANX, APOA2, APOA1, CALR, APOB |
| 43 | GOTERM_CC_DIRECT | GO:0035748~myelin sheath abaxonal region | 2 | 0.0350396 | SCRIB, PRKCZ |
| 44 | GOTERM_CC_DIRECT | GO:0005938~cell cortex | 4 | 0.0367207 | FGB, FGA, FGG, ARHGEF7 |
| 45 | GOTERM_CC_DIRECT | GO:0031982~vesicle | 4 | 0.0405645 | CETP, TF, AKT1, PRKCZ |
| 46 | GOTERM_CC_DIRECT | GO:0044233~ER-mitochondrion membrane contact site | 2 | 0.0464469 | CANX, TOMM20 |
| 47 | GOTERM_CC_DIRECT | GO:0031232~extrinsic component of external side of plasma membrane | 2 | 0.0464469 | CUBN, TF |
| 48 | GOTERM_CC_DIRECT | GO:0005769~early endosome | 5 | 0.0473721 | TF, APOA2, APOC3, APOA1, APOB |

**Supplementary Table S7:** Gene ontology enrichment based on molecular functions term for merged networks nodes based on significant P-Value.

|  | Category | Term | Count | PValue | Genes |
| --- | --- | --- | --- | --- | --- |
| 1 | GOTERM_MF_DIRECT | GO:0031994~insulin-like growth factor I binding | 9 | 8.26E-16 | IGFBP1, IGFBP5, IGFBP4, ITGB3, INSR, IGFBP3, IGFBP2, IGFBP6, IGF1R |
| 2 | GOTERM_MF_DIRECT | GO:0031995~insulin-like growth factor II binding | 7 | 1.39E-12 | IGFBP1, IGFBP5, IGFBP4, INSR, IGFBP3, IGFBP2, IGFBP6 |
| 3 | GOTERM_MF_DIRECT | GO:0005515~protein binding | 80 | 8.37E-07 | SCARB1, DYRK4, NUP107, ITGAM, TFRC, ITGB3, ITGB2, HP, HBB, TOMM20, CDC73, IGF1R, ICAM1, SERPINA5, ADAMTS4, CDC20, PCMT1, YWHAQ, TUBB3, AKT1, FBXO5, LBP, MBL2, SKP1, DSP, FGB, FGA, CUBN, IGFBP5, MIOS, IGFBP3, PRKCE, KRT1, IGFBP2, FGG, APOA2, ZFP69B, KRT7, APOA1, SCRIB, KRT5, ACTA1, RBP4, TFR2, CANX, LRIF1, ARHGEF7, LPA, SEH1L, NAXE, NPRL2, LCAT, LRP2, LPP, PRKCZ, WDR24, KNG1, C3, VTN, ATP5B, FZR1, EXOSC10, TTR, NUP85, SH3BP2, STX4, APOB, APOL1, EVI5, ABCA1, CD163, SMAD3, INSR, F12, TF, TRIP6, ALB, CALR, CD22, TJP2 |
| 4 | GOTERM_MF_DIRECT | GO:0017127~cholesterol transporter activity | 5 | 1.83E-06 | ABCA1, CETP, APOA2, APOA1, APOB |
| 5 | GOTERM_MF_DIRECT | GO:0005102~receptor binding | 13 | 1.98E-06 | FGB, ABCA1, IGFBP1, FGA, IGFBP4, FGG, IGFBP2, KNG1, C3, APOF, IGFBP6, LBP, MBL2 |
| 6 | GOTERM_MF_DIRECT | GO:0015485~cholesterol binding | 6 | 5.31E-06 | ABCA1, CETP, APOA2, APOF, APOC3, APOA1 |
| 7 | GOTERM_MF_DIRECT | GO:0031210~phosphatidylcholine binding | 5 | 1.14E-05 | CETP, APOA2, APOA1, RPE65, SERPINA5 |
| 8 | GOTERM_MF_DIRECT | GO:0001968~fibronectin binding | 5 | 1.90E-05 | IGFBP5, ITGB3, IGFBP3, FBLN1, LPA |
| 9 | GOTERM_MF_DIRECT | GO:0005198~structural molecule activity | 10 | 2.35E-05 | FGB, DSP, FGA, KRT27, MAP7D1, KRT1, FGG, KRT7, KRT5, EVPL |
| 10 | GOTERM_MF_DIRECT | GO:0043560~insulin receptor substrate binding | 4 | 3.72E-05 | INSR, INSRR, PRKCZ, IGF1R |
| 11 | GOTERM_MF_DIRECT | GO:0050839~cell adhesion molecule binding | 6 | 4.13E-05 | FGB, DSP, FGA, ITGB3, ITGB2, FGG |
| 12 | GOTERM_MF_DIRECT | GO:0001948~glycoprotein binding | 6 | 5.20E-05 | ITGAM, TFRC, TFR2, CANX, ITGB2, CALR |
| 13 | GOTERM_MF_DIRECT | GO:0034185~apolipoprotein binding | 4 | 8.10E-05 | ABCA1, SCARB1, CANX, LPA |
| 14 | GOTERM_MF_DIRECT | GO:0034186~apolipoprotein A-I binding | 3 | 1.14E-04 | ABCA1, SCARB1, LCAT |
| 15 | GOTERM_MF_DIRECT | GO:0070653~high-density lipoprotein particle receptor binding | 3 | 1.14E-04 | APOA2, APOC3, APOA1 |
| 16 | GOTERM_MF_DIRECT | GO:0030674~protein binding, bridging | 6 | 1.17E-04 | FGB, DSP, FGA, FGG, EVPL, TJP2 |
| 17 | GOTERM_MF_DIRECT | GO:0005543~phospholipid binding | 6 | 1.98E-04 | ABCA1, PON1, APOA2, APOC3, APOA1, APOB |
| 18 | GOTERM_MF_DIRECT | GO:0030492~hemoglobin binding | 3 | 2.28E-04 | HP, HBB, HPR |
| 19 | GOTERM_MF_DIRECT | GO:0005319~lipid transporter activity | 4 | 2.47E-04 | CETP, APOA2, APOF, APOB |
| 20 | GOTERM_MF_DIRECT | GO:0071889~14-3-3 protein binding | 4 | 4.30E-04 | YWHAQ, PRKCE, AKT1, PRKCZ |
| 21 | GOTERM_MF_DIRECT | GO:0055102~lipase inhibitor activity | 3 | 5.66E-04 | APOA2, APOC3, APOA1 |
| 22 | GOTERM_MF_DIRECT | GO:0008035~high-density lipoprotein particle binding | 3 | 0.001047 | SCARB1, APOA2, APOA1 |
| 23 | GOTERM_MF_DIRECT | GO:0005548~phospholipid transporter activity | 3 | 0.001669 | ABCA1, CETP, APOA1 |
| 24 | GOTERM_MF_DIRECT | GO:0008289~lipid binding | 6 | 0.002525 | CETP, APOA2, APOC3, APOA1, APOL1, PLTP |
| 25 | GOTERM_MF_DIRECT | GO:0004252~serine-type endopeptidase activity | 7 | 0.005192 | C3, F12, C1RL, HP, HPR, LPA, MBL2 |
| 26 | GOTERM_MF_DIRECT | GO:0005520~insulin-like growth factor binding | 3 | 0.005494 | IGFBP1, IGFBP3, IGF1R |
| 27 | GOTERM_MF_DIRECT | GO:0043548~phosphatidylinositol 3-kinase binding | 3 | 0.006116 | INSR, INSRR, IGF1R |
| 28 | GOTERM_MF_DIRECT | GO:0017022~myosin binding | 3 | 0.008162 | ACTA1, ACTC1, STX4 |
| 29 | GOTERM_MF_DIRECT | GO:0001618~virus receptor activity | 4 | 0.009467 | SCARB1, TFRC, ITGB3, ICAM1 |
| 30 | GOTERM_MF_DIRECT | GO:0004998~transferrin receptor activity | 2 | 0.012402 | TFRC, TFR2 |
| 31 | GOTERM_MF_DIRECT | GO:0008201~heparin binding | 5 | 0.017746 | VTN, APOB, LPA, KNG1, SERPINA5 |
| 32 | GOTERM_MF_DIRECT | GO:0034190~apolipoprotein receptor binding | 2 | 0.018545 | APOA2, APOA1 |
| 33 | GOTERM_MF_DIRECT | GO:0004714~transmembrane receptor protein tyrosine kinase activity | 3 | 0.023292 | INSR, INSRR, IGF1R |
| 34 | GOTERM_MF_DIRECT | GO:0005178~integrin binding | 4 | 0.027756 | VTN, FBLN1, CALR, ICAM1 |
| 35 | GOTERM_MF_DIRECT | GO:0043559~insulin binding | 2 | 0.030719 | INSR, IGF1R |
| 36 | GOTERM_MF_DIRECT | GO:0005200~structural constituent of cytoskeleton | 4 | 0.031257 | DSP, ACTA1, TUBB3, KRT5 |
| 37 | GOTERM_MF_DIRECT | GO:0030246~carbohydrate binding | 5 | 0.034034 | CANX, KRT1, CALR, CD22, MBL2 |
| 38 | GOTERM_MF_DIRECT | GO:0005509~calcium ion binding | 10 | 0.034548 | CALB2, CUBN, F12, PON1, CANX, FBLN1, LRP2, CALR, NID1, MBL2 |
| 39 | GOTERM_MF_DIRECT | GO:0010997~anaphase-promoting complex binding | 2 | 0.03675 | CDC20, FZR1 |
| 40 | GOTERM_MF_DIRECT | GO:0060228~phosphatidylcholine-sterol O-acyltransferase activator activity | 2 | 0.03675 | APOA2, APOA1 |
| 41 | GOTERM_MF_DIRECT | GO:0005215~transporter activity | 5 | 0.037379 | ABCA1, SCARB1, ATP5B, CUBN, RBP4 |
| 42 | GOTERM_MF_DIRECT | GO:0042802~identical protein binding | 10 | 0.043635 | VTN, SMAD3, TTR, TFRC, ITGB3, ALB, APOA1, AKT1, FBLN1, IGF1R |
| 43 | GOTERM_MF_DIRECT | GO:0004872~receptor activity | 5 | 0.046542 | CUBN, ITGB3, KRT1, ITGB2, ICAM1 |
| 44 | GOTERM_MF_DIRECT | GO:0097027~ubiquitin-protein transferase activator activity | 2 | 0.0487 | CDC20, FZR1 |

**Supplementary Table S8:** The enriched GO analysis of most important module of PPI network based on significant P-Value.

|  | | Category | | Term | | Count | | P-Value | | Genes | |
| --- | --- | --- | --- | --- | --- | --- | --- | --- | --- | --- | --- |
| 1 | | GOTERM_BP_DIRECT | | GO:0001523~retinoid metabolic process | | 5 | | 56+A2:F12 | | RBP4, APOA2, APOC3, APOA1, APOB | |
| 2 | | GOTERM_BP_DIRECT | | GO:0002576~platelet degranulation | | 5 | | 2.73E-07 | | FGB, FGA, TF, FGG, APOA1 | |
| 3 | | GOTERM_BP_DIRECT | | GO:0033344~cholesterol efflux | | 4 | | 3.47E-07 | | APOA2, APOC3, APOA1, APOB | |
| 4 | | GOTERM_BP_DIRECT | | GO:1900026~positive regulation of substrate adhesion-dependent cell spreading | | 4 | | 7.48E-07 | | FGB, FGA, FGG, APOA1 | |
| 5 | | GOTERM_BP_DIRECT | | GO:0010903~negative regulation of very-low-density lipoprotein particle remodeling | | 3 | | 9.57E-07 | | APOA2, APOC3, APOA1 | |
| 6 | | GOTERM_BP_DIRECT | | GO:0042157~lipoprotein metabolic process | | 4 | | 1.27E-06 | | APOA2, APOC3, APOA1, APOB | |
| 7 | | GOTERM_BP_DIRECT | | GO:0072378~blood coagulation, fibrin clot formation | | 3 | | 1.91E-06 | | FGB, FGA, FGG | |
| 8 | | GOTERM_BP_DIRECT | | GO:0042632~cholesterol homeostasis | | 4 | | 6.22E-06 | | APOA2, APOC3, APOA1, APOB | |
| 9 | | GOTERM_BP_DIRECT | | GO:0090277~positive regulation of peptide hormone secretion | | 3 | | 8.92E-06 | | FGB, FGA, FGG | |
| 10 | | GOTERM_BP_DIRECT | | GO:0031639~plasminogen activation | | 3 | | 1.15E-05 | | FGB, FGA, FGG | |
| 11 | | GOTERM_BP_DIRECT | | GO:0042158~lipoprotein biosynthetic process | | 3 | | 1.15E-05 | | APOA2, APOA1, APOB | |
| 12 | | GOTERM_BP_DIRECT | | GO:0034116~positive regulation of heterotypic cell-cell adhesion | | 3 | | 1.75E-05 | | FGB, FGA, FGG | |
| 13 | | GOTERM_BP_DIRECT | | GO:0051258~protein polymerization | | 3 | | 2.48E-05 | | FGB, FGA, FGG | |
| 14 | | GOTERM_BP_DIRECT | | GO:0033700~phospholipid efflux | | 3 | | 2.89E-05 | | APOA2, APOC3, APOA1 | |
| 15 | | GOTERM_BP_DIRECT | | GO:0034375~high-density lipoprotein particle remodeling | | 3 | | 3.34E-05 | | APOA2, APOC3, APOA1 | |
| 16 | | GOTERM_BP_DIRECT | | GO:0043691~reverse cholesterol transport | | 3 | | 4.86E-05 | | APOA2, APOC3, APOA1 | |
| 17 | | GOTERM_BP_DIRECT | | GO:0043623~cellular protein complex assembly | | 3 | | 4.86E-05 | | FGB, FGA, FGG | |
| 18 | | GOTERM_BP_DIRECT | | GO:0045921~positive regulation of exocytosis | | 3 | | 6.66E-05 | | FGB, FGA, FGG | |
| 19 | | GOTERM_BP_DIRECT | | GO:0042730~fibrinolysis | | 3 | | 6.66E-05 | | FGB, FGA, FGG | |
| 20 | | GOTERM_BP_DIRECT | | GO:0019433~triglyceride catabolic process | | 3 | | 9.51E-05 | | APOC3, APOA1, APOB | |
| 21 | | GOTERM_BP_DIRECT | | GO:2000352~negative regulation of endothelial cell apoptotic process | | 3 | | 1.20E-04 | | FGB, FGA, FGG | |
| 22 | | GOTERM_BP_DIRECT | | GO:0045907~positive regulation of vasoconstriction | | 3 | | 1.57E-04 | | FGB, FGA, FGG | |
| 23 | | GOTERM_BP_DIRECT | | GO:1902042~negative regulation of extrinsic apoptotic signaling pathway via death domain receptors | | 3 | | 1.67E-04 | | FGB, FGA, FGG | |
| 24 | | GOTERM_BP_DIRECT | | GO:0050714~positive regulation of protein secretion | | 3 | | 1.99E-04 | | FGB, FGA, FGG | |
| 25 | | GOTERM_BP_DIRECT | | GO:0070527~platelet aggregation | | 3 | | 2.59E-04 | | FGB, FGA, FGG | |
| 26 | | GOTERM_BP_DIRECT | | GO:0051592~response to calcium ion | | 3 | | 5.18E-04 | | FGB, FGA, FGG | |
| 27 | | GOTERM_BP_DIRECT | | GO:0008203~cholesterol metabolic process | | 3 | | 7.12E-04 | | APOA2, APOA1, APOB | |
| 28 | | GOTERM_BP_DIRECT | | GO:0006869~lipid transport | | 3 | | 8.89E-04 | | APOA2, APOC3, APOA1 | |
| 29 | | GOTERM_BP_DIRECT | | GO:0060621~negative regulation of cholesterol import | | 2 | | 0.001191 | | APOA2, APOC3 | |
| 30 | | GOTERM_BP_DIRECT | | GO:0007160~cell-matrix adhesion | | 3 | | 0.001243 | | FGB, FGA, FGG | |
| 31 | | GOTERM_BP_DIRECT | | GO:0018158~protein oxidation | | 2 | | 0.001786 | | APOA2, APOA1 | |
| 32 | | GOTERM_BP_DIRECT | | GO:0030168~platelet activation | | 3 | | 0.002019 | | FGB, FGA, FGG | |
| 33 | | GOTERM_BP_DIRECT | | GO:0060192~negative regulation of lipase activity | | 2 | | 0.00238 | | APOA2, APOA1 | |
| 34 | | GOTERM_BP_DIRECT | | GO:0043152~induction of bacterial agglutination | | 2 | | 0.00238 | | FGB, FGA | |
| 35 | | GOTERM_BP_DIRECT | | GO:0051180~vitamin transport | | 2 | | 0.002974 | | APOA1, GC | |
| 36 | | GOTERM_BP_DIRECT | | GO:0032489~regulation of Cdc42 protein signal transduction | | 2 | | 0.002974 | | APOC3, APOA1 | |
| 37 | | GOTERM_BP_DIRECT | | GO:0030300~regulation of intestinal cholesterol absorption | | 2 | | 0.002974 | | APOA2, APOA1 | |
| 38 | | GOTERM_BP_DIRECT | | GO:0018206~peptidyl-methionine modification | | 2 | | 0.003568 | | APOA2, APOA1 | |
| 39 | | GOTERM_BP_DIRECT | | GO:0002740~negative regulation of cytokine secretion involved in immune response | | 2 | | 0.003568 | | APOA2, APOA1 | |
| 40 | | GOTERM_BP_DIRECT | | GO:0034384~high-density lipoprotein particle clearance | | 2 | | 0.003568 | | APOA2, APOA1 | |
| 41 | | GOTERM_BP_DIRECT | | GO:0070374~positive regulation of ERK1 and ERK2 cascade | | 3 | | 0.0046 | | FGB, FGA, FGG | |
| 42 | | GOTERM_BP_DIRECT | | GO:0034380~high-density lipoprotein particle assembly | | 2 | | 0.004755 | | APOA2, APOA1 | |
| 43 | | GOTERM_BP_DIRECT | | GO:0034379~very-low-density lipoprotein particle assembly | | 2 | | 0.004755 | | APOC3, APOB | |
| 44 | | GOTERM_BP_DIRECT | | GO:0007596~blood coagulation | | 3 | | 0.005072 | | FGB, FGA, FGG | |
| 45 | | GOTERM_BP_DIRECT | | GO:0006898~receptor-mediated endocytosis | | 3 | | 0.00518 | | HP, APOA1, APOB | |
| 46 | | GOTERM_BP_DIRECT | | GO:0010873~positive regulation of cholesterol esterification | | 2 | | 0.005348 | | APOA2, APOA1 | |
| 47 | | GOTERM_BP_DIRECT | | GO:0030198~extracellular matrix organization | | 3 | | 0.005735 | | FGB, FGA, FGG | |
| 48 | | GOTERM_BP_DIRECT | | GO:0034374~low-density lipoprotein particle remodeling | | 2 | | 0.006533 | | APOA2, APOB | |
| 49 | | GOTERM_BP_DIRECT | | GO:0050995~negative regulation of lipid catabolic process | | 2 | | 0.00949 | | APOA2, APOC3 | |
| 50 | | GOTERM_BP_DIRECT | | GO:0030301~cholesterol transport | | 2 | | 0.00949 | | APOA1, APOB | |
| 51 | | GOTERM_BP_DIRECT | | GO:0006656~phosphatidylcholine biosynthetic process | | 2 | | 0.014793 | | APOA2, APOA1 | |
| 52 | | GOTERM_BP_DIRECT | | GO:0070328~triglyceride homeostasis | | 2 | | 0.01538 | | APOC3, APOA1 | |
| 53 | | GOTERM_BP_DIRECT | | GO:0006641~triglyceride metabolic process | | 2 | | 0.020654 | | APOA2, APOC3 | |
| 54 | | GOTERM_BP_DIRECT | | GO:0031100~organ regeneration | | 2 | | 0.027647 | | APOA2, APOA1 | |
| 55 | | GOTERM_BP_DIRECT | | GO:0043627~response to estrogen | | 2 | | 0.038052 | | APOA2, APOA1 | |
| 56 | | GOTERM_BP_DIRECT | | GO:0031647~regulation of protein stability | | 2 | | 0.040924 | | TF, APOA2 | |
|  | | **Cellular component** | | | | | | | | | |
| 1 | | GOTERM_CC_DIRECT | GO:0072562~blood microparticle | | 8 | | 2.87E-13 | | FGB, FGA, TF, FGG, APOA2, HP, APOA1, GC | |  |
| 2 | | GOTERM_CC_DIRECT | GO:0005576~extracellular region | | | 11 | | 2.82E-11 | | FGB, FGA, TF, RBP4, FGG, APOA2, HP, APOC3, APOA1, APOB, GC | |
| 3 | | GOTERM_CC_DIRECT | GO:0005615~extracellular space | | | 10 | | 6.00E-10 | | FGB, FGA, TF, RBP4, FGG, HP, APOC3, APOA1, APOB, GC | |
| 4 | | GOTERM_CC_DIRECT | GO:0070062~extracellular exosome | | | 11 | | 7.52E-09 | | FGB, FGA, TF, RBP4, FGG, APOA2, HP, APOC3, APOA1, APOB, GC | |
| 5 | | GOTERM_CC_DIRECT | GO:0042627~chylomicron | | | 4 | | 4.32E-08 | | APOA2, APOC3, APOA1, APOB | |
| 6 | | GOTERM_CC_DIRECT | GO:0034361~very-low-density lipoprotein particle | | | 4 | | 1.35E-07 | | APOA2, APOC3, APOA1, APOB | |
| 7 | | GOTERM_CC_DIRECT | GO:0005769~early endosome | | | 5 | | 4.81E-06 | | TF, APOA2, APOC3, APOA1, APOB | |
| 8 | | GOTERM_CC_DIRECT | GO:0034366~spherical high-density lipoprotein particle | | | 3 | | 7.57E-06 | | APOA2, APOC3, APOA1 | |
| 9 | | GOTERM_CC_DIRECT | GO:0005577~fibrinogen complex | | | 3 | | 9.74E-06 | | FGB, FGA, FGG | |
| 10 | | GOTERM_CC_DIRECT | GO:0031091~platelet alpha granule | | | 3 | | 2.46E-05 | | FGB, FGA, FGG | |
| 11 | | GOTERM_CC_DIRECT | GO:0071682~endocytic vesicle lumen | | | 3 | | 3.24E-05 | | HP, APOA1, APOB | |
| 12 | | GOTERM_CC_DIRECT | GO:0009986~cell surface | | | 5 | | 1.41E-04 | | FGB, FGA, TF, FGG, APOA1 | |
| 13 | | GOTERM_CC_DIRECT | GO:1903561~extracellular vesicle | | | 3 | | 3.27E-04 | | FGB, FGA, APOA1 | |
| 14 | | GOTERM_CC_DIRECT | GO:0031093~platelet alpha granule lumen | | | 3 | | 3.96E-04 | | FGB, FGA, FGG | |
| 15 | | GOTERM_CC_DIRECT | GO:0005938~cell cortex | | | 3 | | 0.001963 | | FGB, FGA, FGG | |
| 16 | | GOTERM_CC_DIRECT | GO:0034363~intermediate-density lipoprotein particle | | | 2 | | 0.002193 | | APOC3, APOB | |
| 17 | | GOTERM_CC_DIRECT | GO:0005788~endoplasmic reticulum lumen | | | 3 | | 0.0047 | | APOA2, APOA1, APOB | |
| 18 | | GOTERM_CC_DIRECT | GO:0009897~external side of plasma membrane | | | 3 | | 0.005752 | | FGB, FGA, FGG | |
| 19 | | GOTERM_CC_DIRECT | GO:0034774~secretory granule lumen | | | 2 | | 0.006567 | | TF, APOA1 | |
| 20 | | GOTERM_CC_DIRECT | GO:0034364~high-density lipoprotein particle | | | 2 | | 0.01201 | | APOA2, APOA1 | |
| 21 | | GOTERM_CC_DIRECT | GO:0030139~endocytic vesicle | | | 2 | | 0.029246 | | TF, APOA1 | |
|  | | **Molecular Functions** | | | | | | | | | |
| 1 | GOTERM_MF_DIRECT | | GO:0070653~high-density lipoprotein particle receptor binding | | | 3 | | 9.47E-07 | | APOA2, APOC3, APOA1 | |
| 2 | GOTERM_MF_DIRECT | | GO:0055102~lipase inhibitor activity | | | 3 | | 4.73E-06 | | APOA2, APOC3, APOA1 | |
| 3 | GOTERM_MF_DIRECT | | GO:0005543~phospholipid binding | | | 4 | | 1.49E-05 | | APOA2, APOC3, APOA1, APOB | |
| 4 | GOTERM_MF_DIRECT | | GO:0017127~cholesterol transporter activity | | | 3 | | 3.30E-05 | | APOA2, APOA1, APOB | |
| 5 | GOTERM_MF_DIRECT | | GO:0015485~cholesterol binding | | | 3 | | 2.56E-04 | | APOA2, APOC3, APOA1 | |
| 6 | GOTERM_MF_DIRECT | | GO:0050839~cell adhesion molecule binding | | | 3 | | 5.86E-04 | | FGB, FGA, FGG | |
| 7 | GOTERM_MF_DIRECT | | GO:0030674~protein binding, bridging | | | 3 | | 9.03E-04 | | FGB, FGA, FGG | |
| 8 | GOTERM_MF_DIRECT | | GO:0034190~apolipoprotein receptor binding | | | 2 | | 0.001776 | | APOA2, APOA1 | |
| 9 | GOTERM_MF_DIRECT | | GO:0008289~lipid binding | | | 3 | | 0.003412 | | APOA2, APOC3, APOA1 | |
| 10 | GOTERM_MF_DIRECT | | GO:0060228~phosphatidylcholine-sterol O-acyltransferase activator activity | | | 2 | | 0.00355 | | APOA2, APOA1 | |
| 11 | GOTERM_MF_DIRECT | | GO:0008035~high-density lipoprotein particle binding | | | 2 | | 0.00473 | | APOA2, APOA1 | |
| 12 | GOTERM_MF_DIRECT | | GO:0005198~structural molecule activity | | | 3 | | 0.00888 | | FGB, FGA, FGG | |
| 13 | GOTERM_MF_DIRECT | | GO:0005319~lipid transporter activity | | | 2 | | 0.011788 | | APOA2, APOB | |
| 14 | GOTERM_MF_DIRECT | | GO:0031210~phosphatidylcholine binding | | | 2 | | 0.013545 | | APOA2, APOA1 | |
| 15 | GOTERM_MF_DIRECT | | GO:0005102~receptor binding | | | 3 | | 0.017561 | | FGB, FGA, FGG | |
