# Supplementary material for: Proteomic-Based Discovery of Predictive Biomarkers for Drug Therapy Response and Personalized Medicine in Chronic Immune Thrombocytopenia
Source: Biomed Res Int. 2023 Oct 31;2023:9573863. doi: 10.1155/2023/9573863 (PMC10630023; doi:10.1155/2023/9573863)
Supplement: Supplementary 2 — Supplementary figures: Supplementary Figure S1: nineteen differentially expressed protein spots that were identified by Progenesis SameSpots software (2-DE image analysis). Supplementary Figure S2: 2-DE gel image of the control group. Supplementary Figure S3: The spectra of nineteen proteins, which are identified by MALDI‐TOF MS/MS. Supplementary Figure S4: this shows a graphical representation of the Mascot search results for nineteen identified proteins. Peptide mass fingerprints and MS/MS analysis were searched by the Mascot search engine against the SwissProt protein database. The parameters were utilized in the Mascot search engine: trypsin digestion: maximum one missed cleavage; fixed modification: carbamidomethylation (C); peptide mass tolerance: 1.2 Da; and mass value: [M+H]+ and monoisotopic. When the peptide score was above the cutoff value, protein identification was accepted (p < 0.05). Supplementary Figure S5: protein-protein interaction network of 16 differentially expressed proteins in respondents and nonrespondents was retrieved from the STRING database source. The PPI network consists of 16 nodes and 24 edges. Proteins are represented by nodes in the network, and PPI is represented by edges. [file 9573863.f2.docx]

**Proteomic-based discovery of predictive biomarkers for drug therapy response and personalized medicine in chronic immune thrombocytopenia**

**Supplementary figures:**


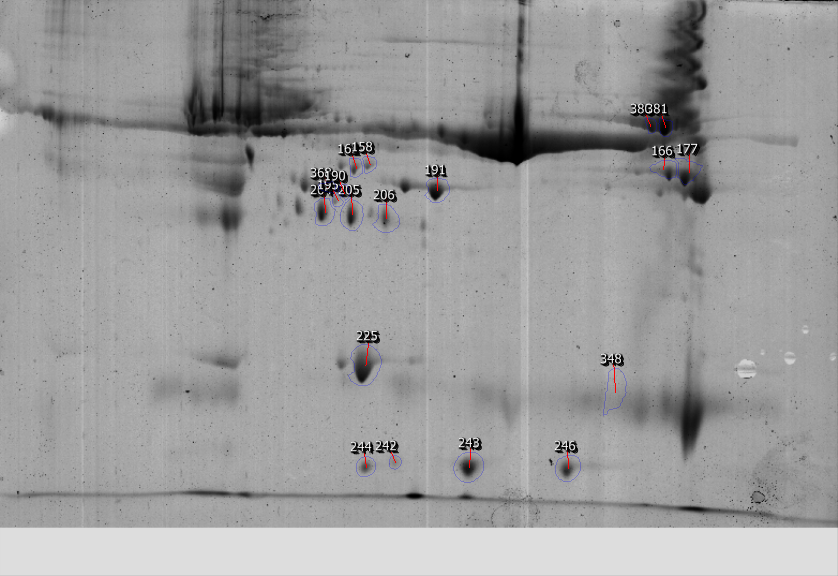


**Supplementary Figure S1:** Nineteen differentially expressed protein spots identified by Progenesis SameSpots software (2-DE image analysis)


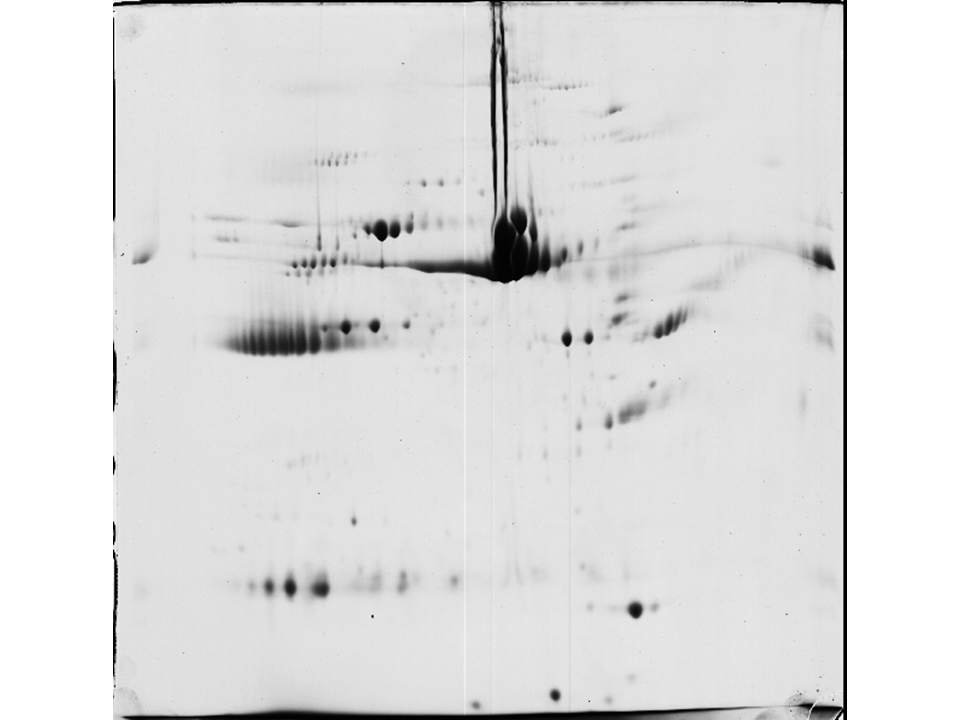


**Supplementary Figure S2**: 2-DE gel image of control group


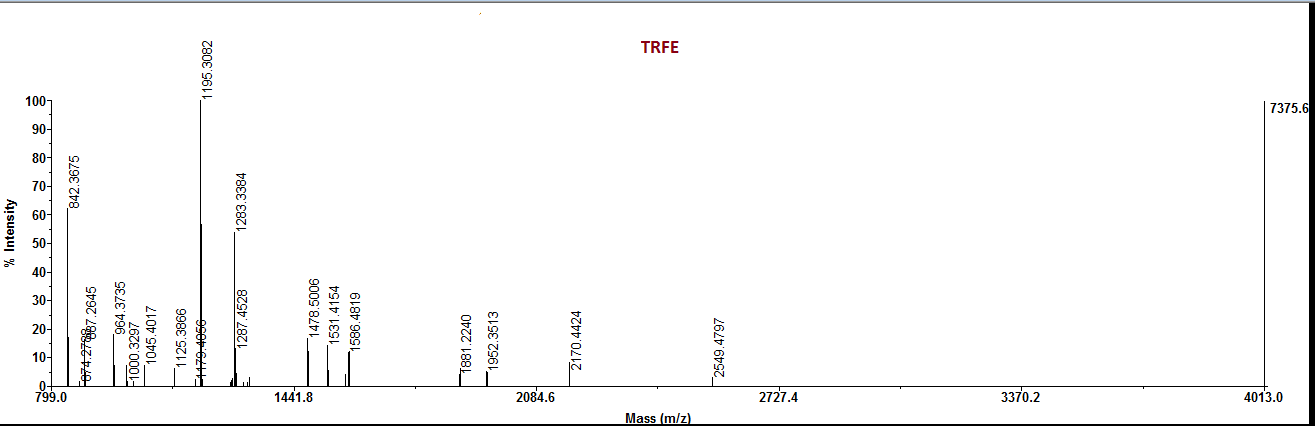


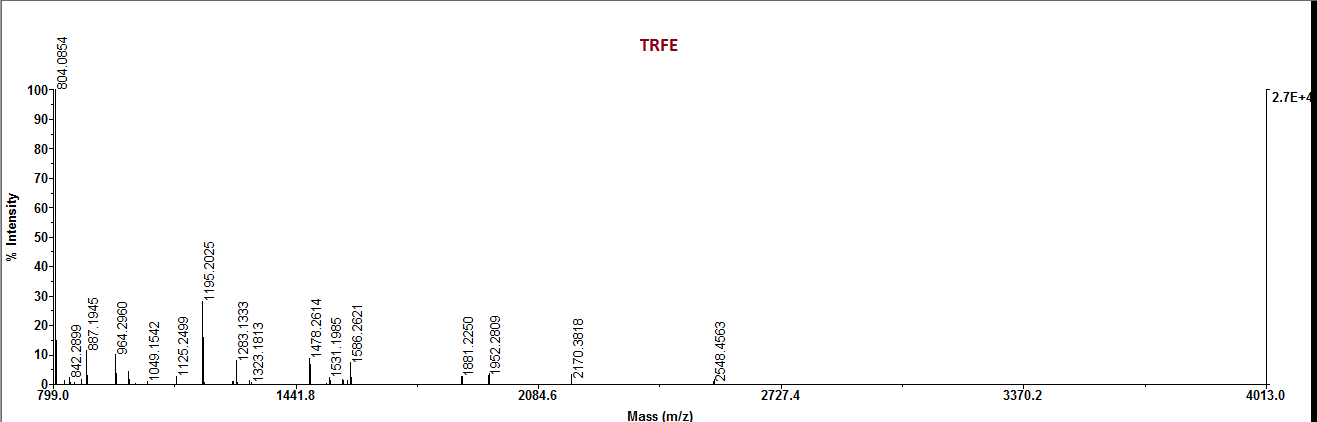

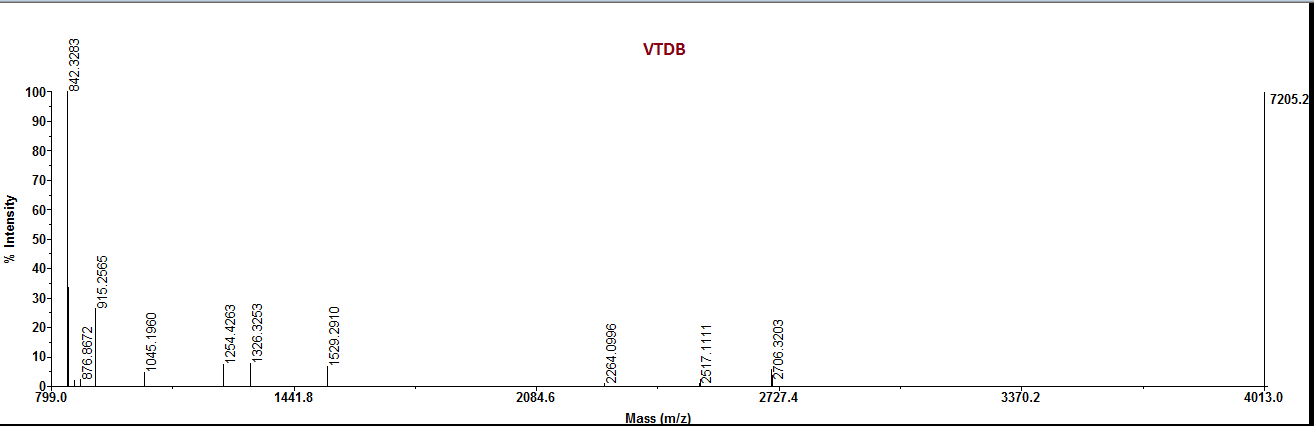

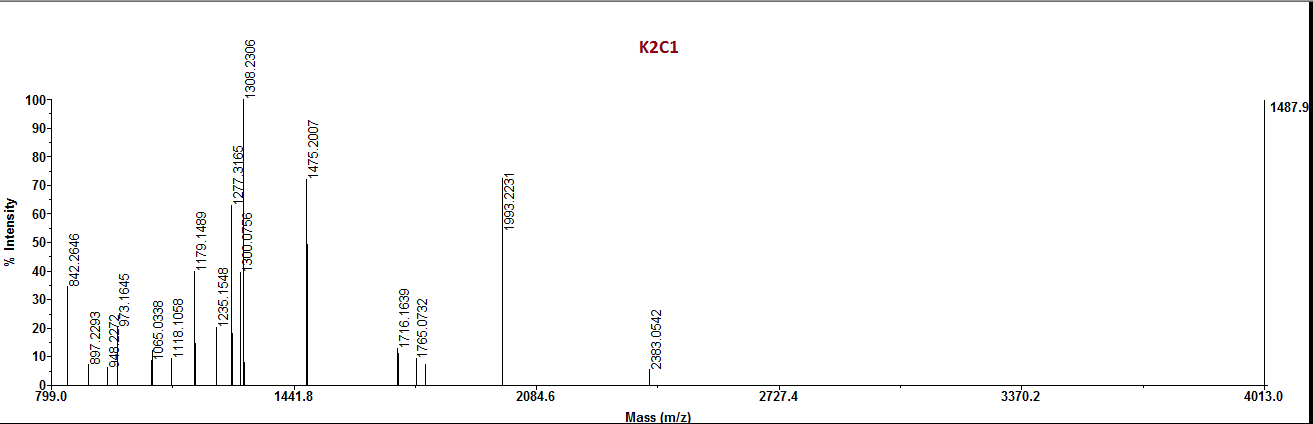

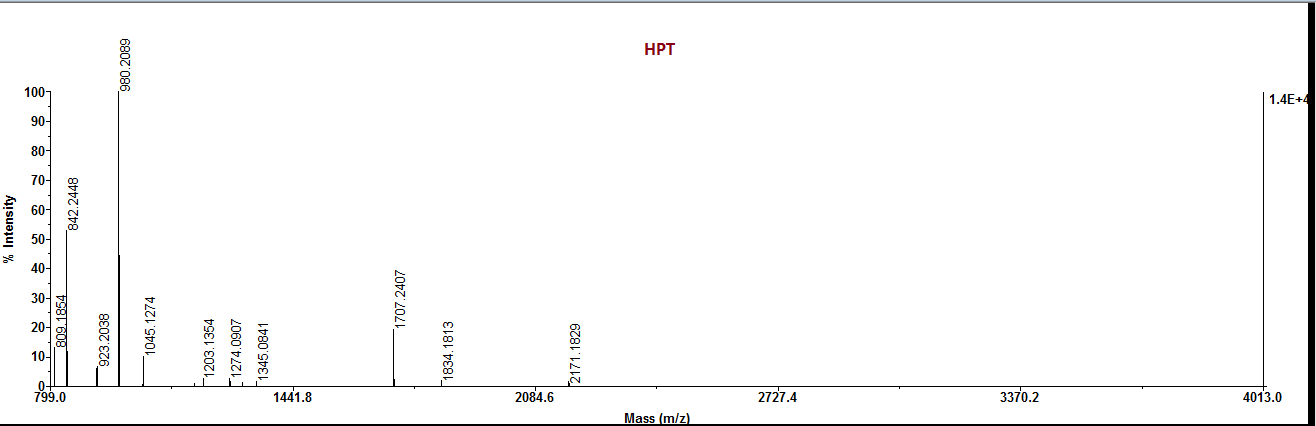

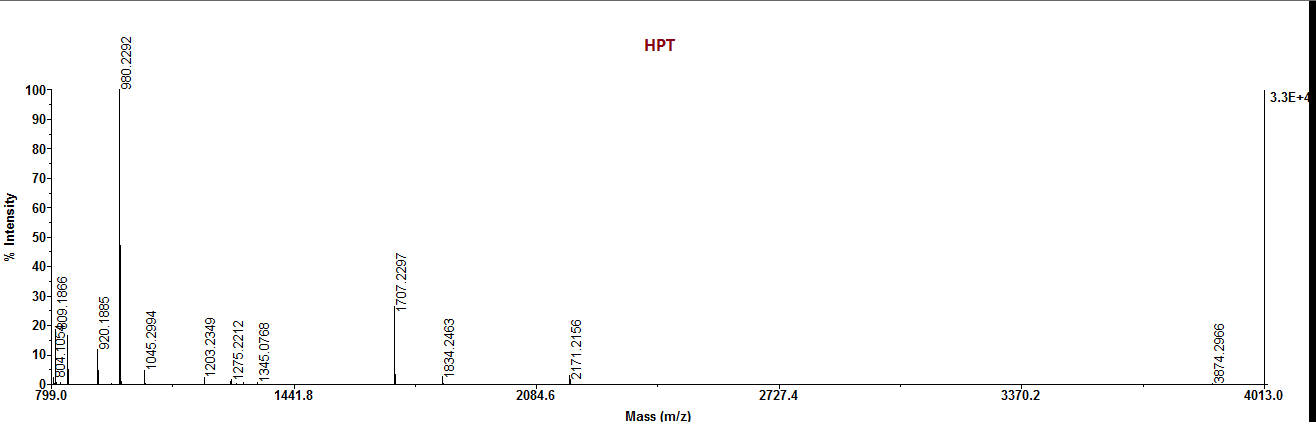

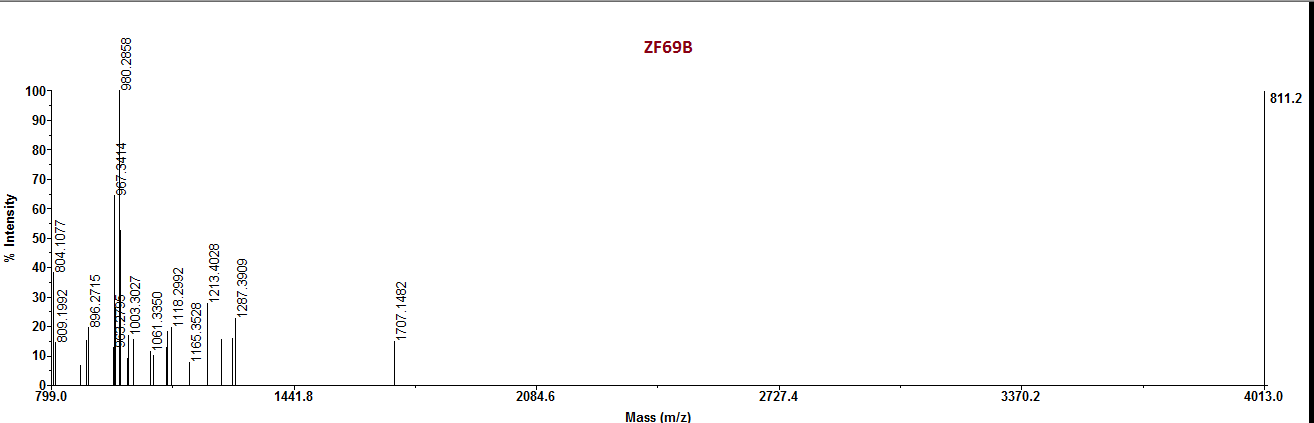

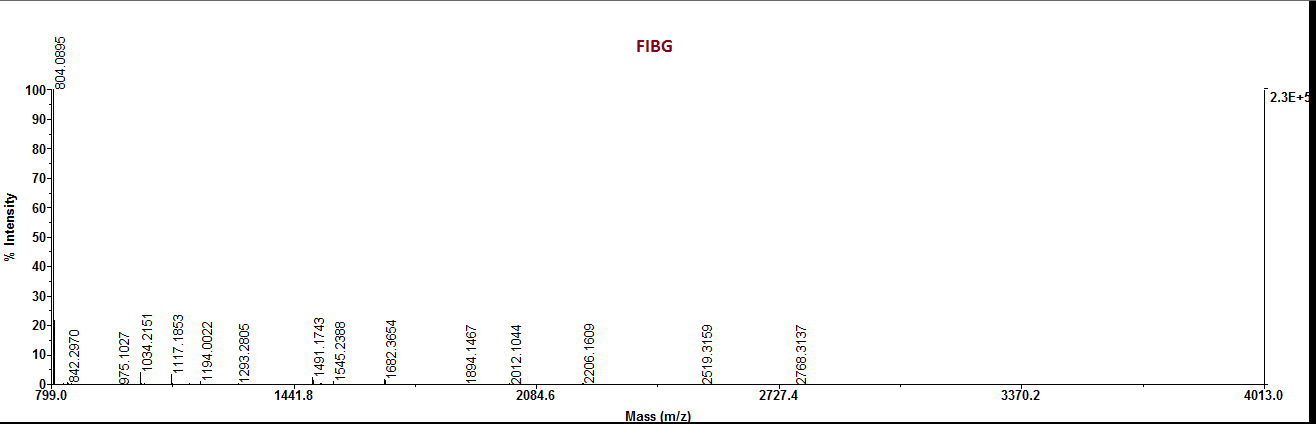

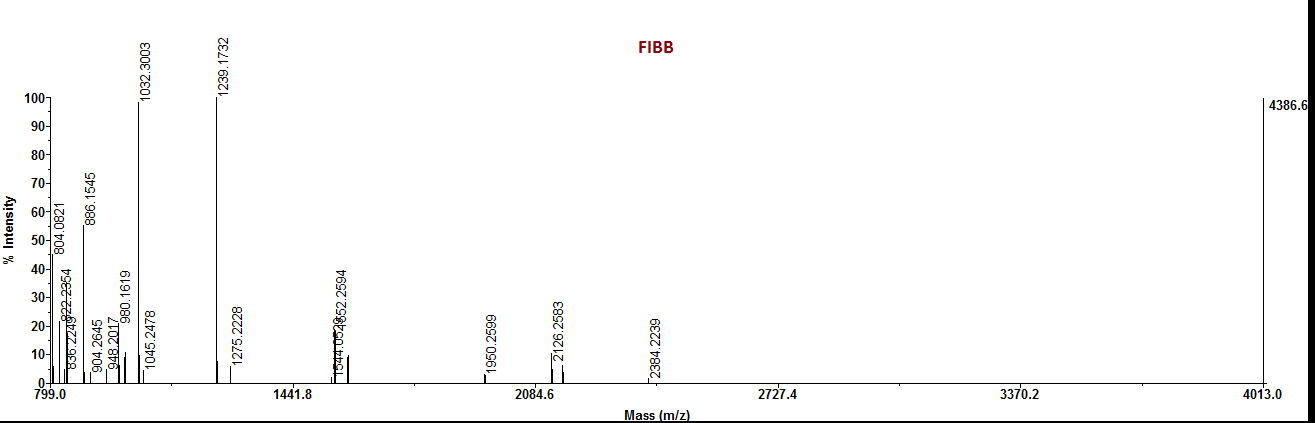

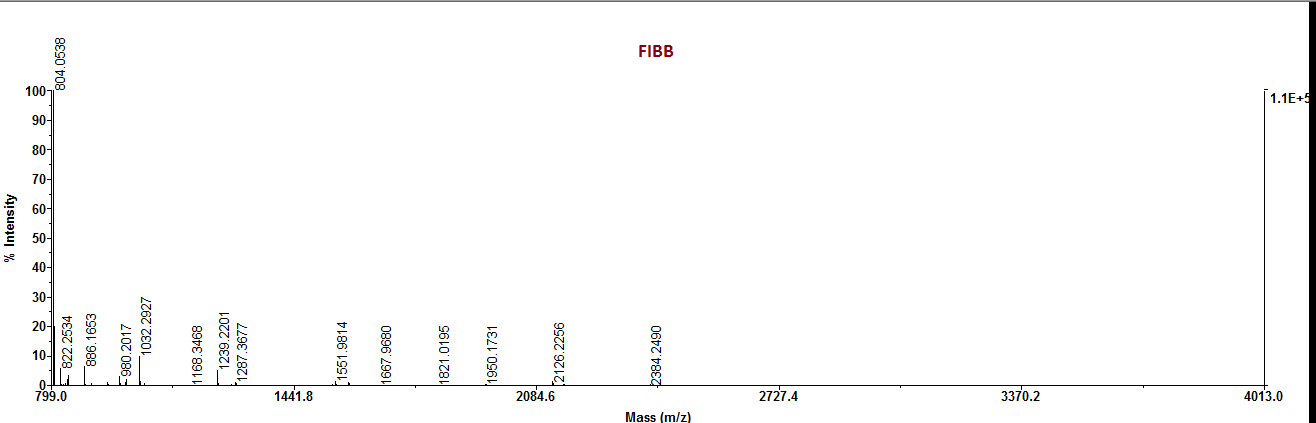

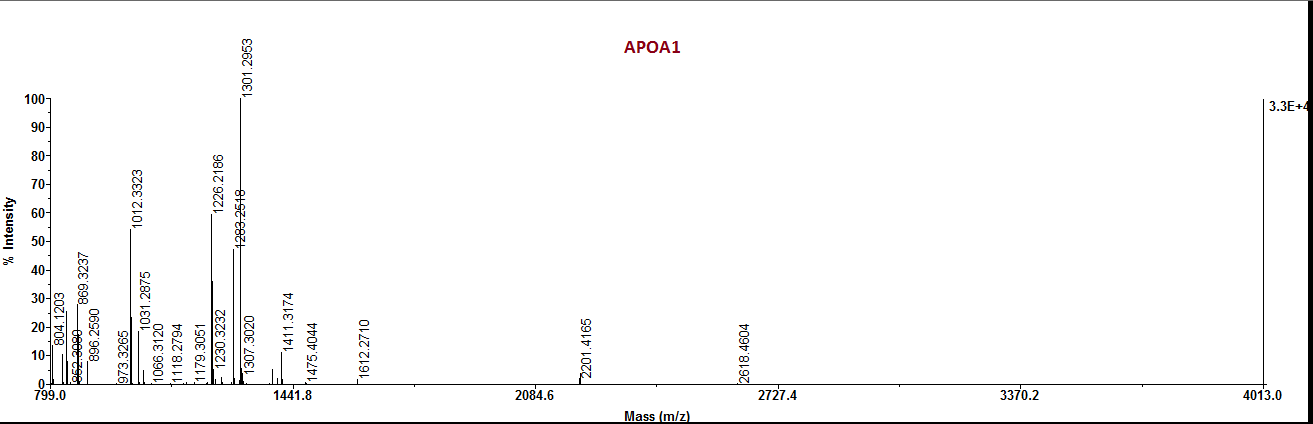

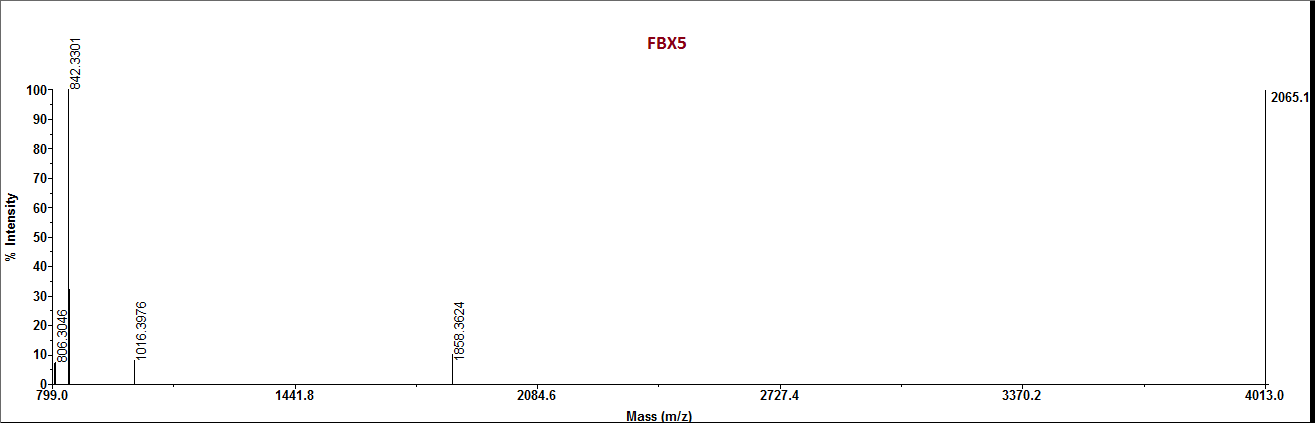

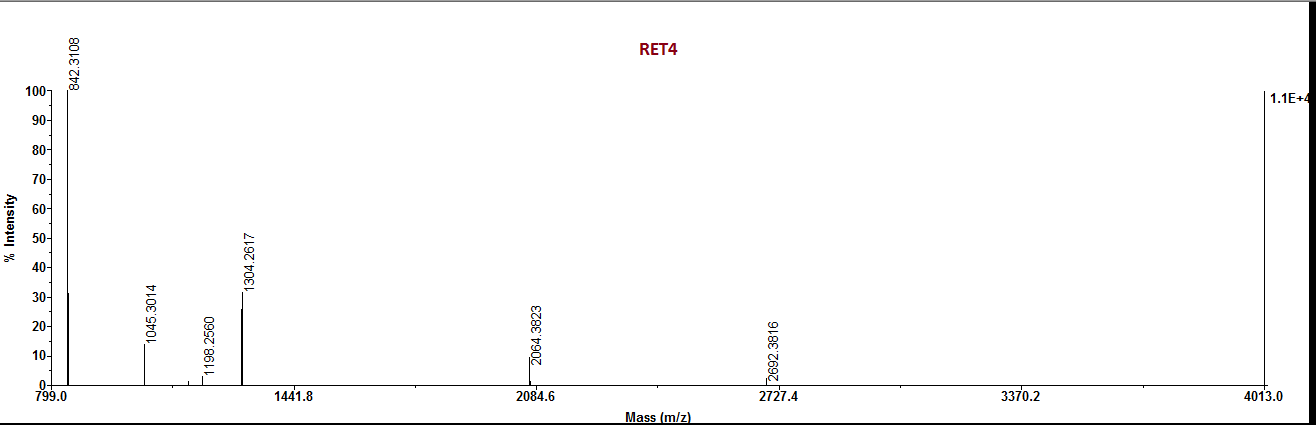

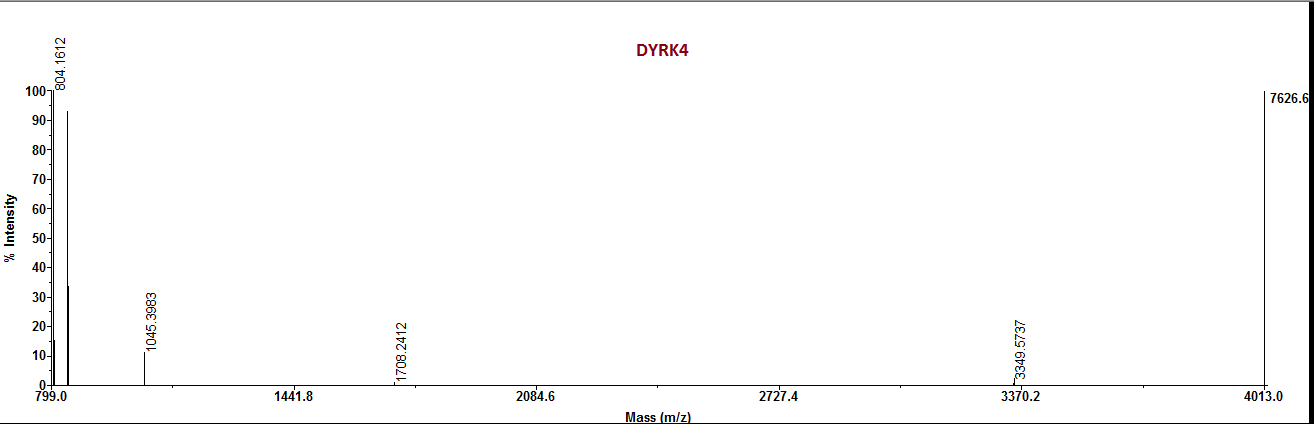

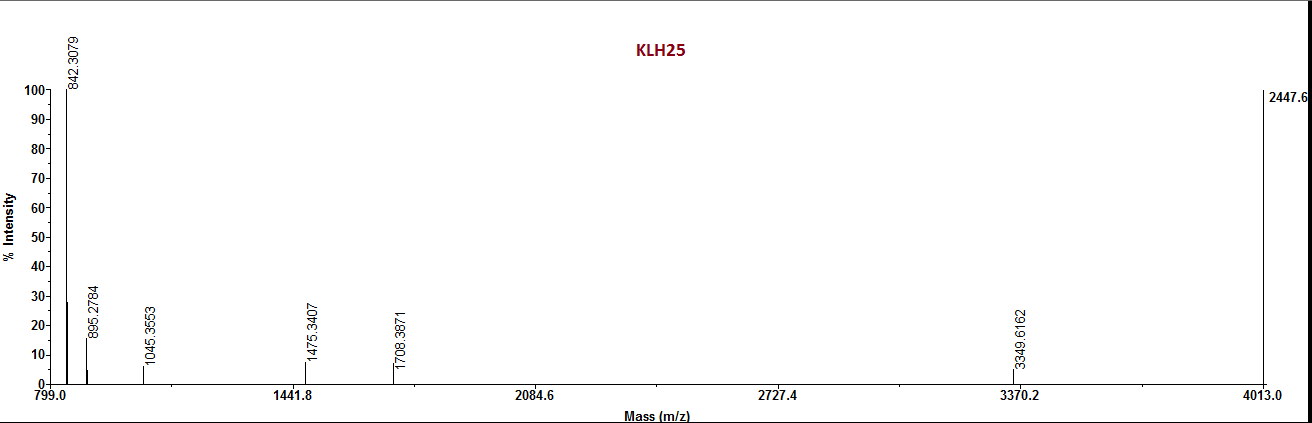

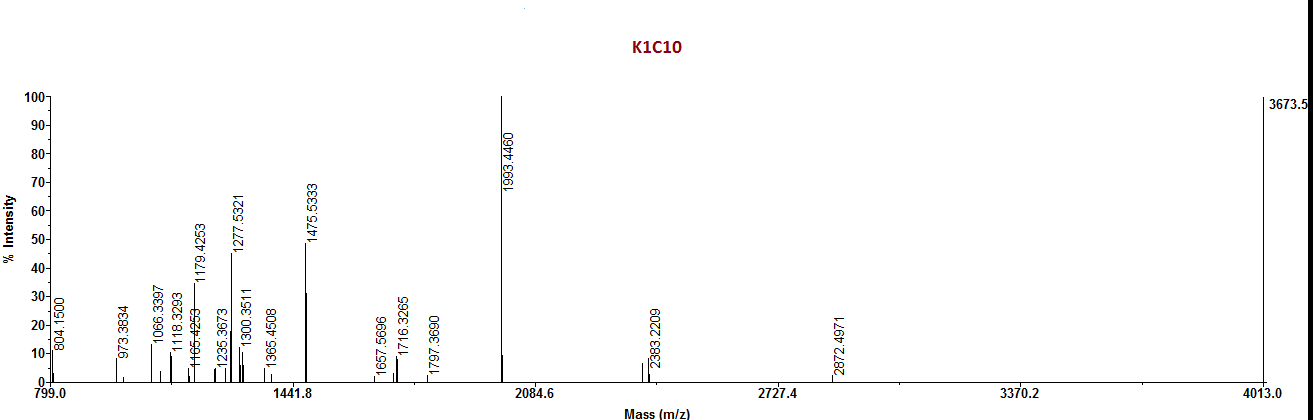

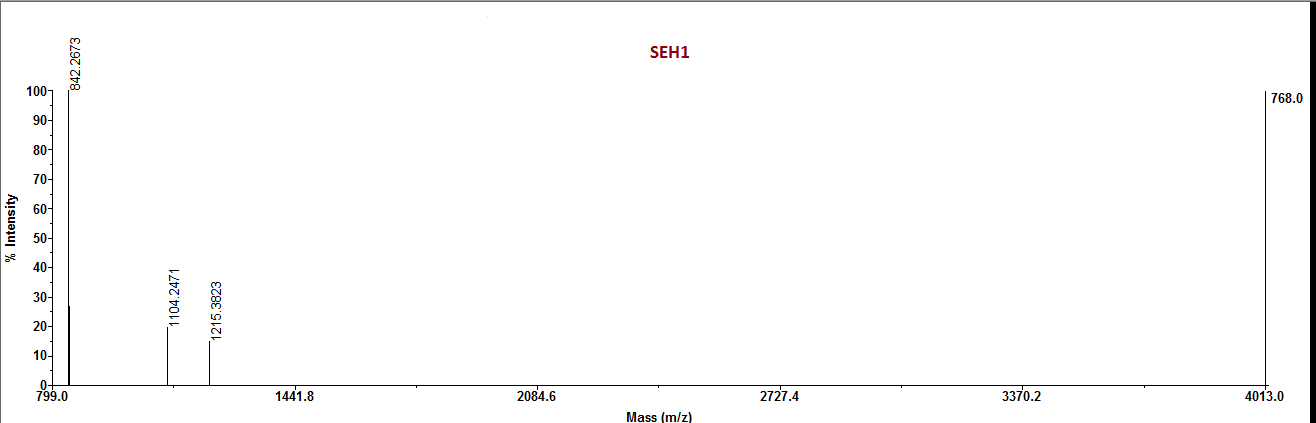

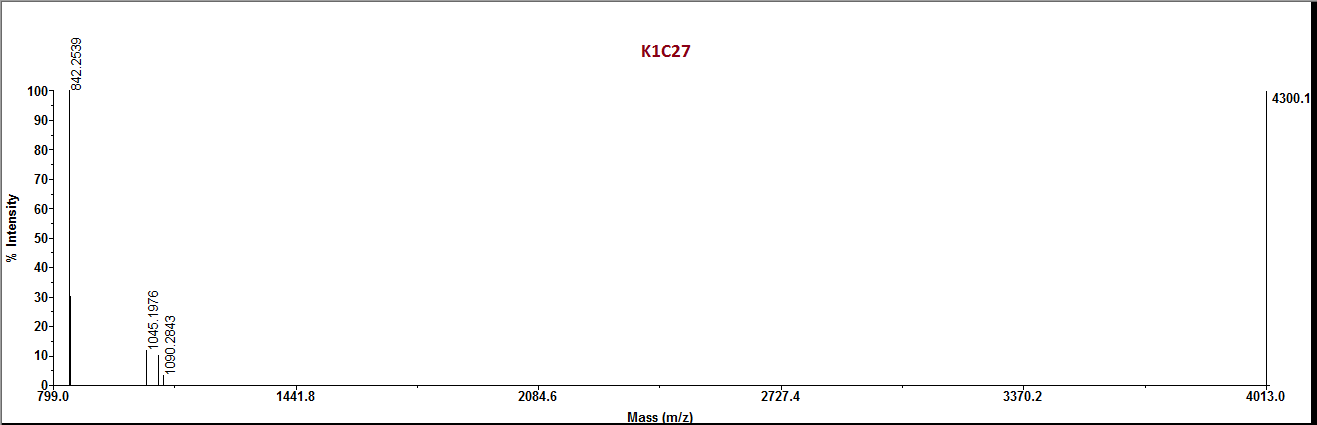

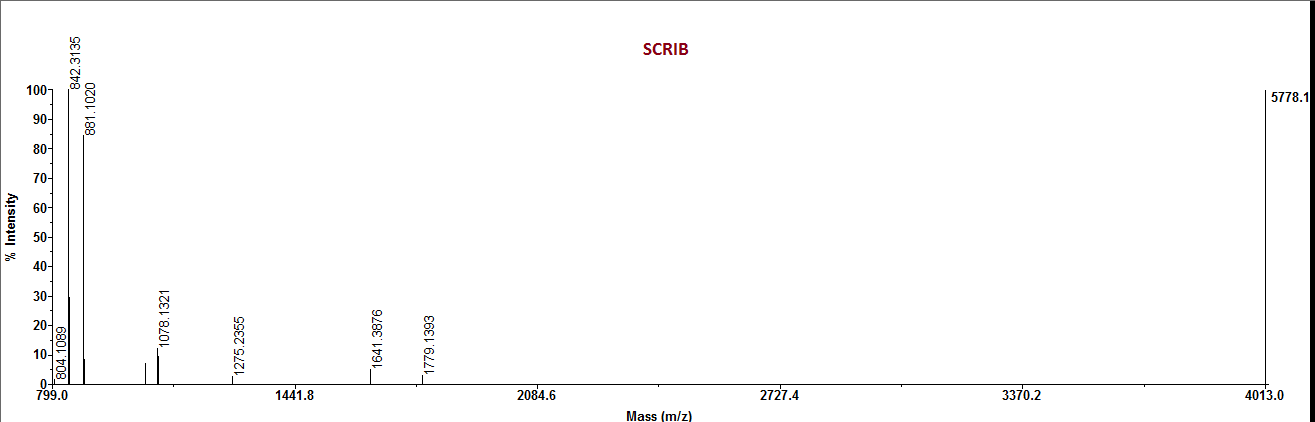


**Supplementary Figure S3**, The spectra of nineteen protein which are identified by MALDI‐TOF MS/MS


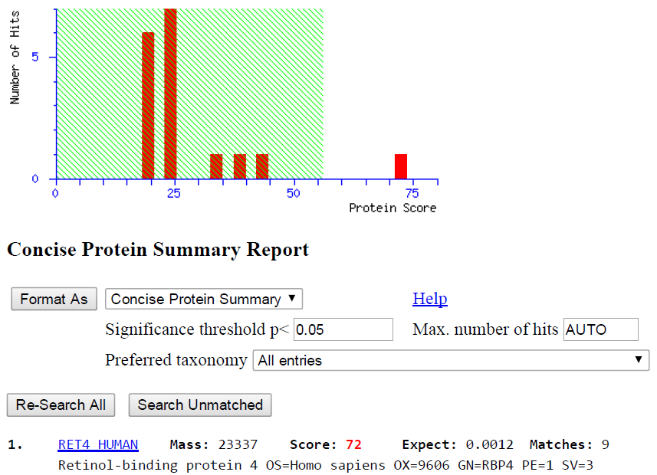

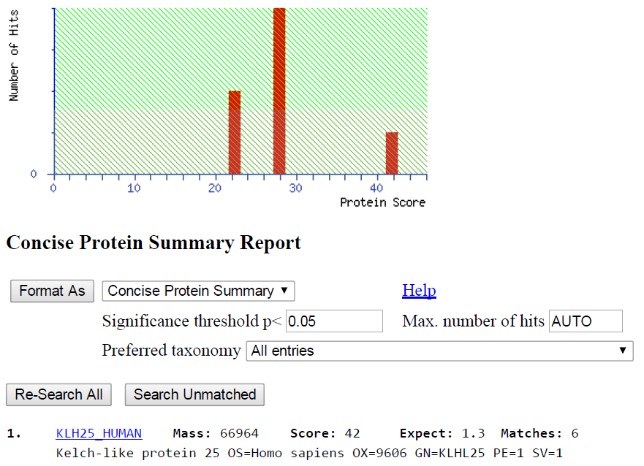

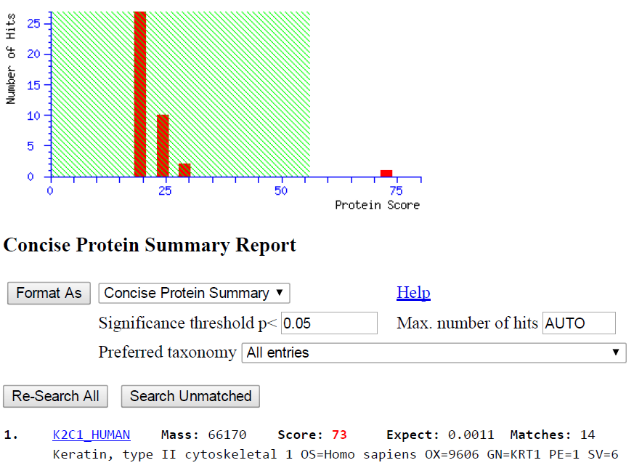

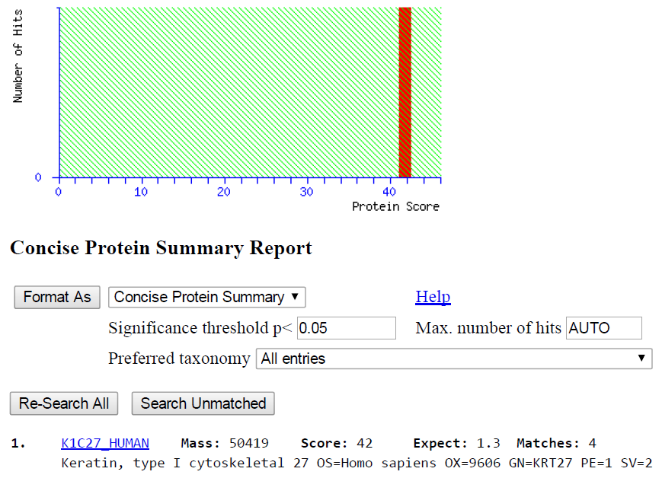

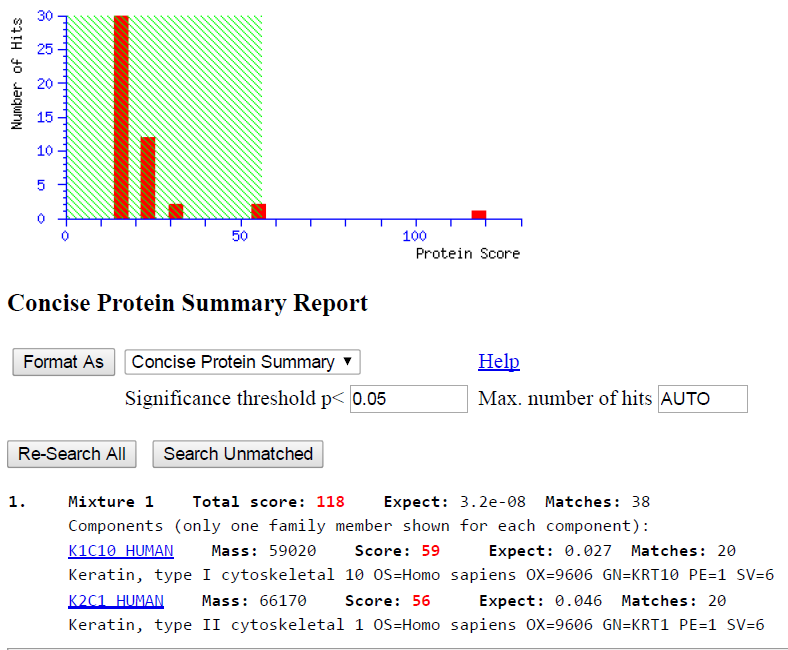

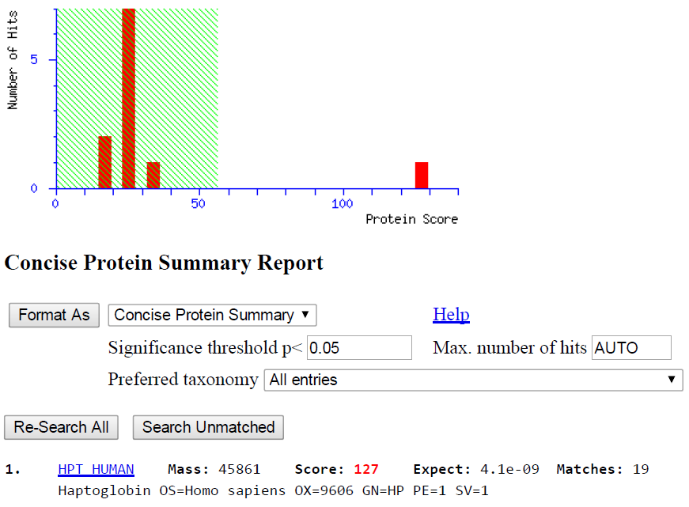

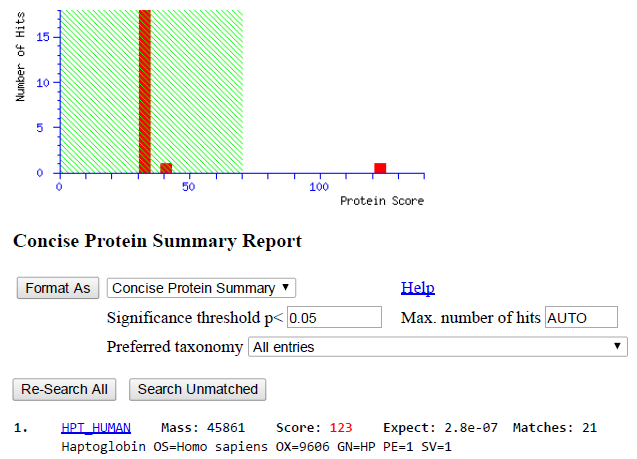

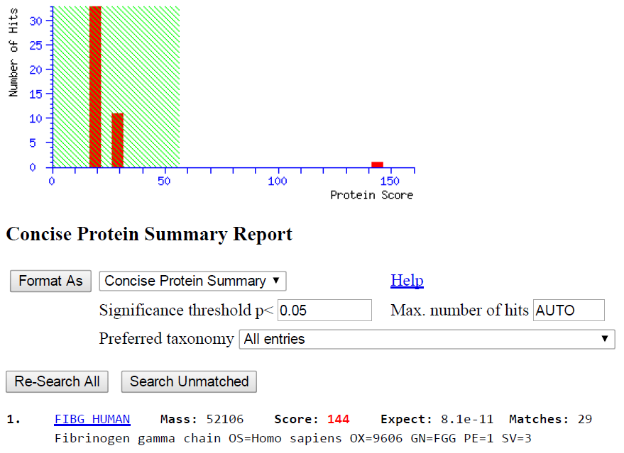

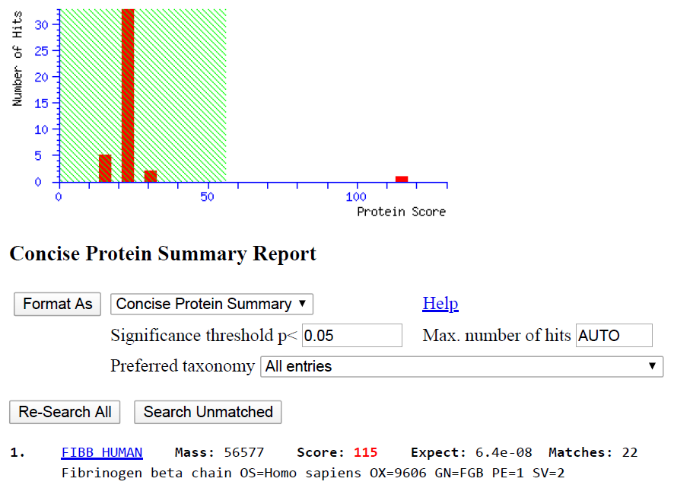

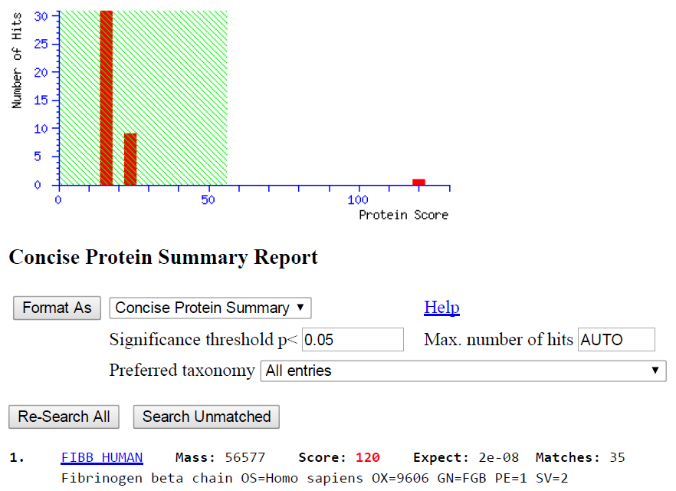

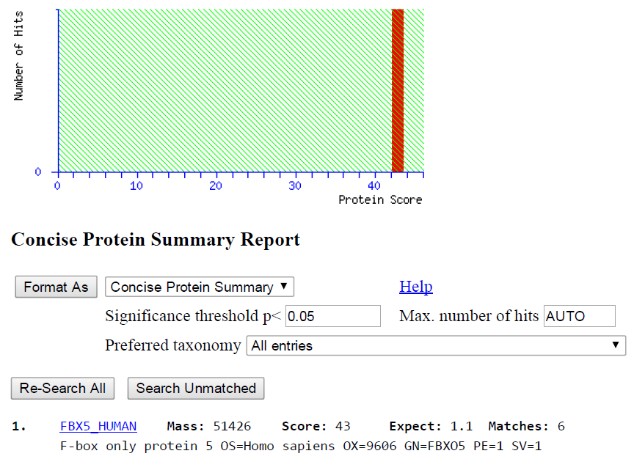

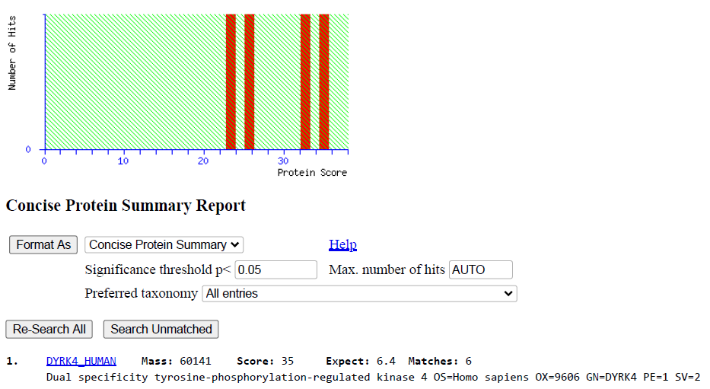

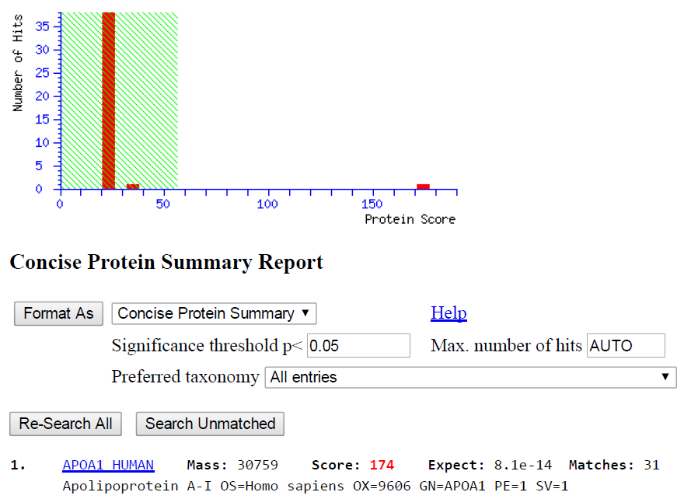

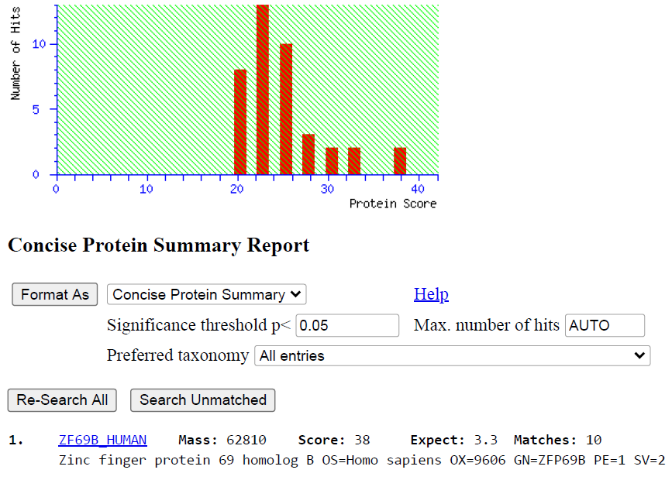

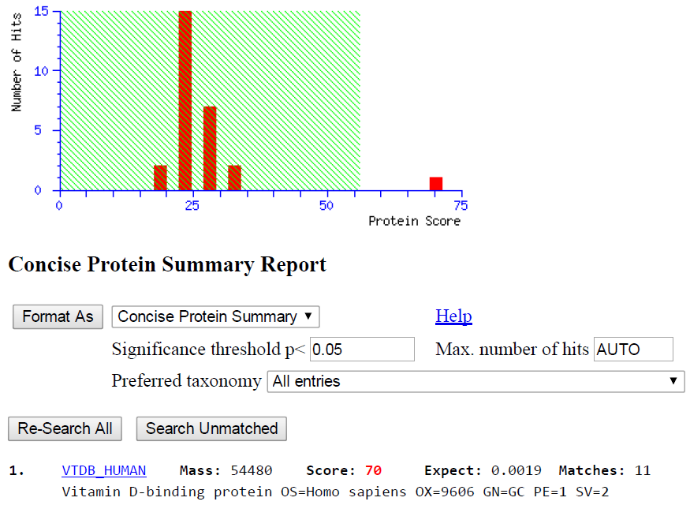

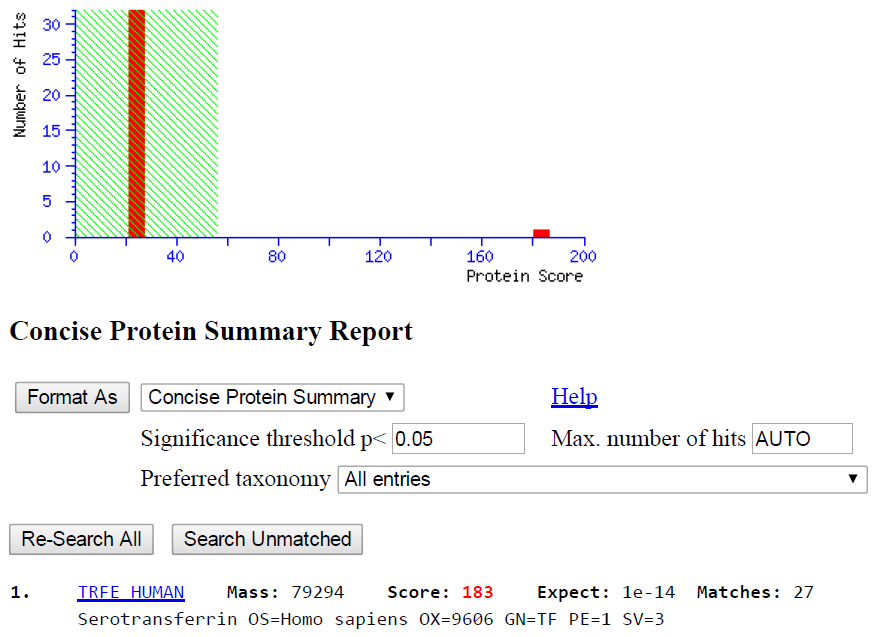

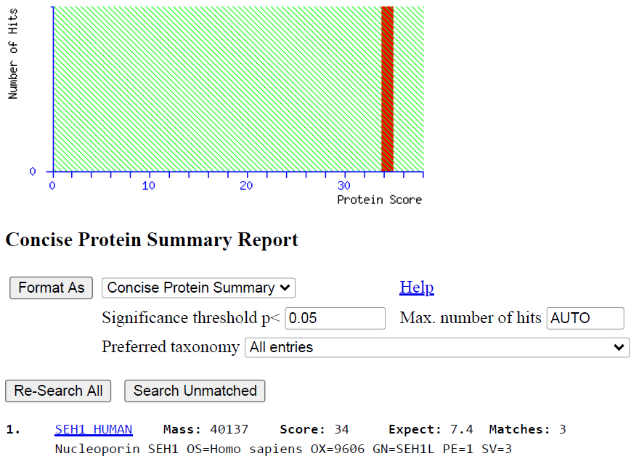

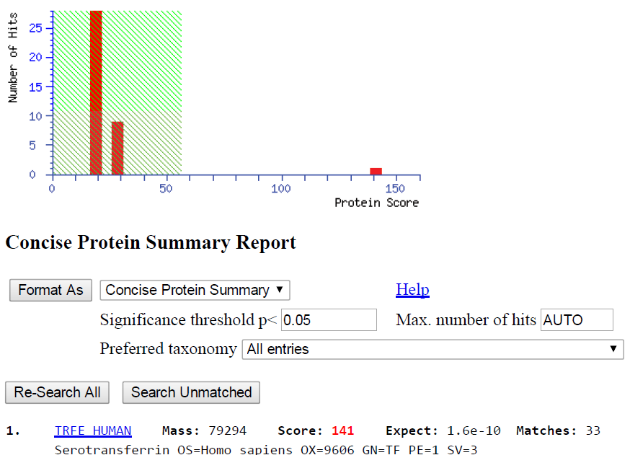

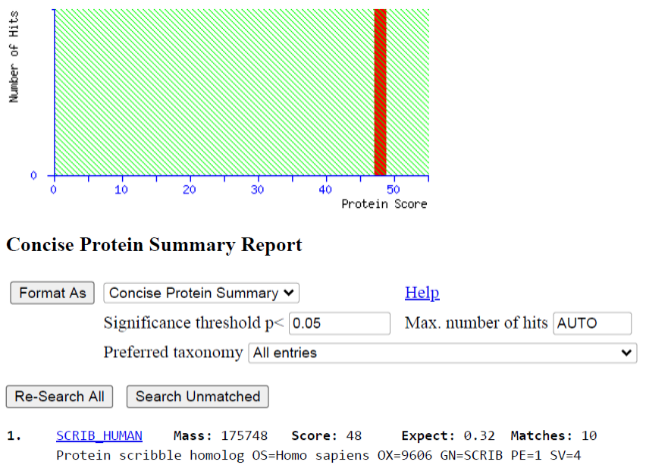


**Supplementary Figure S4.** This shows a graphical representation of the MASCOT search results of nineteen identified protein. Peptide mass fingerprints and MS/MS analysis were searched by the Mascot search engine against the SwissProt protein database. The parameters were utilized in the mascot search engine: Trypsin digestion: maximum one missed cleavage; fixed modification: Carbamidomethylation (C), peptide mass tolerance: 1.2 Da, mass value: [M+H]+ and monoisotopic. When the peptide score was above the cutoff value, protein identification was accepted (p <0.05).


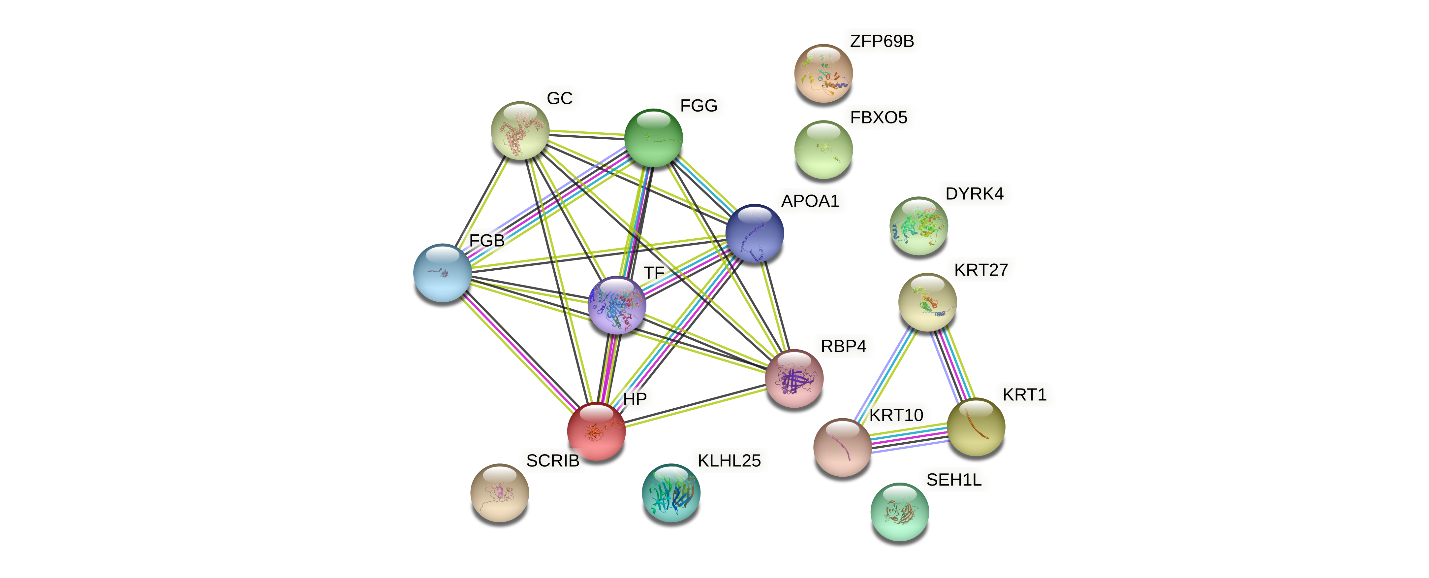


**Supplementary Figure S5.** Protein-protein interaction network of 16 differentially expressed proteins in respondents and non-respondents retrieved from STRING database source. The PPI network consists of 16 nodes and 24 edges. Proteins are represented by nodes in the network, and PPI is represented by edges.
